# Associations of metabolic indicators and inflammation-related indices with adverse cardiovascular events in US adults: NHANES 1999–2018

**DOI:** 10.1186/s12944-026-02866-w

**Published:** 2026-01-21

**Authors:** Shuairong Lin, Jiayue Pan, Xiaoyan Zhu, Ruixu Lan, Xiaojie Sun, Rui Shen, Chuansha Wu

**Affiliations:** 1 School of Medicine, Wuhan University of Science and Technology, Wuhan, 430065 China; 2 Environmental Toxicology Laboratory, Hubei Province Key Laboratory of Occupational Hazard Identification and Control, School of Public Health, Wuhan University of Science and Technology, Wuhan, Hubei China; 3 Healthy Hubei Development and Social Progress Research Center of the Key Research Base of Humanities and Social Sciences in Hubei Province, School of Public Health, Wuhan University of Science and Technology, Wuhan, Hubei China

**Keywords:** Biomarker, Insulin resistance, Inflammation, Cardiovascular disease, Mortality

## Abstract

**Background:**

Processes in cardiovascular disease (CVD) are associated with metabolic perturbations and inflammation. The mediating role of inflammation in connecting metabolic factors to adverse cardiovascular outcomes and the joint effects of both factors on CVD and mortality are poorly understood.

**Methods:**

A total of 18,741 individuals from the National Health and Nutrition Examination Survey 1999–2018 were included in this study. Multivariate survey-weighted logistic regression (for CVD prevalence) and Cox proportional hazards models (for all-cause and CVD mortality) were used to investigate associations among metabolic factors, inflammation-related variables, and adverse cardiovascular outcomes. Mediation analyses were conducted to explore how inflammation may contribute to the relationship between metabolic factors and adverse cardiovascular outcomes. Joint factor models for both metabolic and inflammatory risk factors were developed for comprehensive evaluation.

**Results:**

With each unit rise in the triglyceride–glucose (TyG) index and Dietary Inflammatory Index (DII), the prevalence of CVD and risks of all-cause and CVD mortality escalated by 22% vs. 6% (odds ratio [OR] = 1.22, 95% confidence interval [CI; 1.06, 1.41] vs. 1.06 [1.02, 1.11]), 20% vs. 12% (hazard ratio [HR] = 1.20, 95% CI [1.09, 1.32] vs. 1.12 [1.09, 1.15], and 29% vs. 12% (HR = 1.29, 95% CI [1.01, 1.64] vs. 1.12 [1.05, 1.18]), respectively. Mediation analysis indicated that the DII accounted for 3.58% (95% CI 3.50%, 3.66%) to 17.05% (95% CI 7.77%, 85.09%) of the association of the TyG and atherogenic index of plasma with the prevalence of CVD and risks of all-cause and CVD mortality. Joint effect analysis according to tertiles (T) revealed that compared with the TyG-T1 + DII-T1 group, the TyG-T3 + DII-T3 group had greater CVD prevalence (OR = 1.45, 95% CI [1.02, 2.06]) and elevated risks of all-cause and CVD mortality (HR = 1.80 95% CI [1.47, 2.21]); 1.75 95% CI [1.14, 2.67]).

**Conclusions:**

The study findings revealed that metabolic disturbances and the inflammatory burden have important roles in increasing the prevalence of CVD and risks of all-cause and CVD mortality. Low-cost laboratory and dietary indicators can complement existing assessment, enabling scalable early risk stratification and prioritized primary care intervention to reduce premature CVD deaths across secondary and tertiary prevention.

**Graphical abstract:**

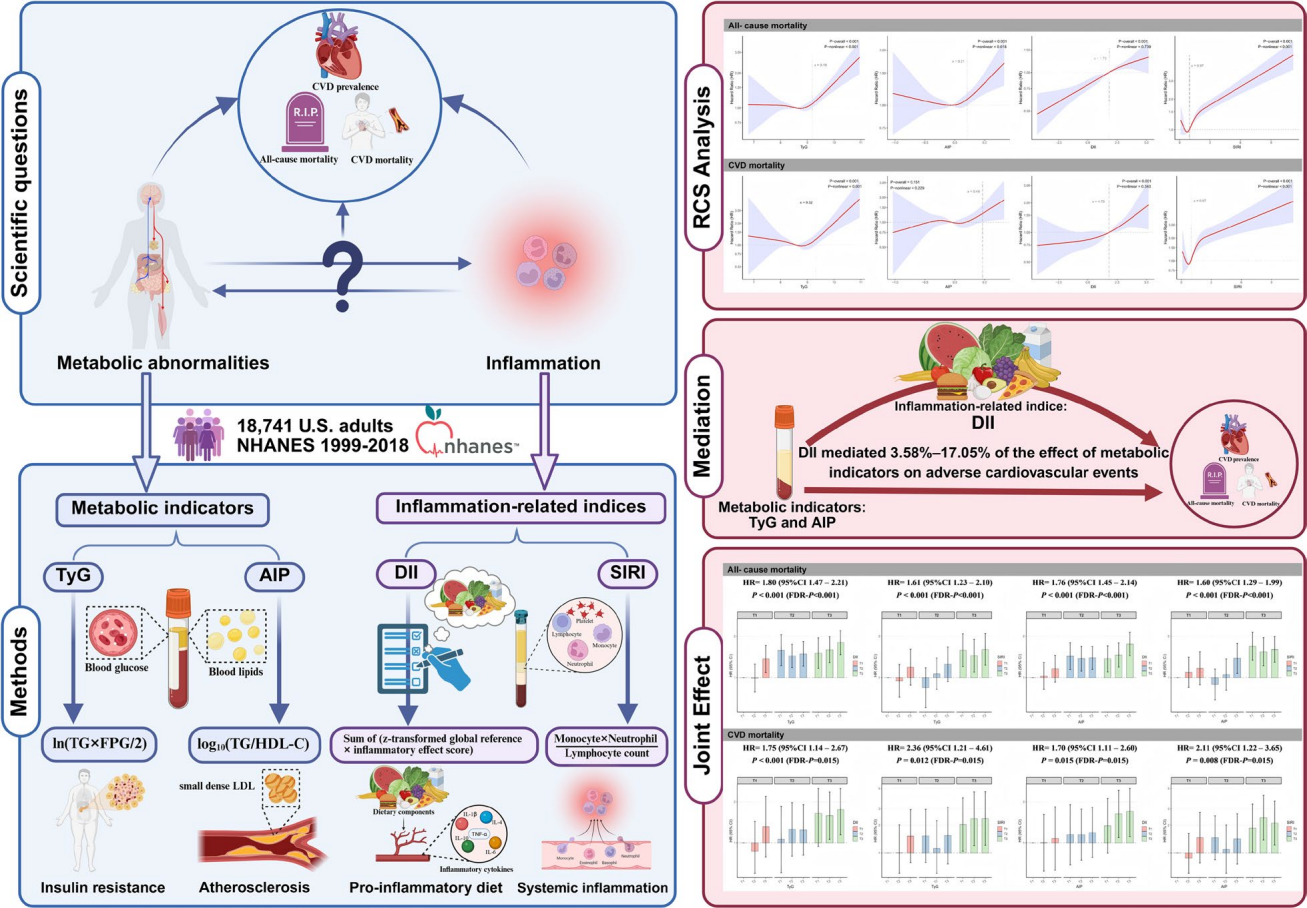

**Supplementary Information:**

The online version contains supplementary material available at 10.1186/s12944-026-02866-w.

## Introduction

Cardiovascular disease (CVD) refers to several diseases of the heart and blood vessels, including heart failure, angina, myocardial infarction, and stroke [1]. CVD remains the leading cause of death worldwide [2, 3], accounting for approximately 20.5 million deaths in 2021, or approximately one-third of all deaths worldwide [4]. Because of this large burden, it is essential to identify and intervene on those factors associated with risks that can be modified so as to decrease morbidity and mortality.

Inflammatory load and metabolic disturbances are both recognized as important risk factors for CVD [5, 6]. Elevated inflammation and metabolic dysregulation are more prevalent among individuals with CVD and are closely associated with increased mortality risk [7, 8]. Chronic inflammation can increase vascular endothelial damage and atherosclerosis via the renin–angiotensin–aldosterone system (RAAS) and other pathways [9]. The dietary inflammatory index (DII) provides a quantitative measure of diet-induced inflammatory changes [10], and the systemic inflammatory response index (SIRI) indicates the overall systemic inflammatory burden [11]. Both indices have been shown to have a significant association with an increased incidence of adverse cardiovascular events [12, 13]. However, the core pathophysiological feature of metabolic abnormalities—insulin resistance (IR)—together with dyslipidemia, jointly drives atherosclerotic changes in large- and medium-sized arteries, thereby accelerating CVD progression [14]. The triglyceride–glucose index (TyG) serves as a convenient surrogate marker for IR [15], and the atherogenic index of plasma (AIP) reflects atherogenic dyslipidemia [16]. Both indices have shown significant associations with unfavorable cardiovascular outcomes. Although IR and dyslipidemia may contribute to chronic inflammation, the mediating, interactive, and joint associations of inflammatory indices and metabolic indices with CVD prevalence and mortality have not been sufficiently explored in large-scale population studies, and more robust evidence is still lacking.

It is hypothesized that inflammation-related indices may mediate, interact with, and jointly amplify the effects of metabolic abnormalities on adverse cardiovascular outcomes. Nationally representative data from the National Health and Nutrition Examination Survey (NHANES) are used to systematically delineate the relationships among metabolic indicators (TyG, AIP), inflammation-related indices (DII, SIRI), CVD prevalence, and mortality. Further analyses examined how inflammation-related indices mediate the relationships between metabolic disorders and both CVD and mortality. Additionally, the interactions and joint effects of combined metabolic and inflammatory indices on these outcomes were assessed. The study aimed to establish a combined risk stratification framework that integrates inflammation and metabolism, including assessment of high-risk thresholds. The objective is to provide references for early screening in community health examinations and outpatient clinics while also offering quantitative evidence for defining high-risk populations in public nutrition policies. An additional objective is to support the implementation of targeted dietary interventions aimed at improving anti-inflammatory and metabolic outcomes.

## Methods

### Study design and study population

This study was based on the NHANES, a project conducted by the United States (U.S.) Centers for Disease Control and Prevention/National Center for Health Statistics (CDC/NCHS). NHANES involves a series of ongoing cross-sectional studies designed to assess health and nutritional status for the past several years, beginning in 1999. The analyses account for NHANES’ multistage probability sampling to produce nationally representative estimates for the non-institutionalized U.S. population. Participants in this study underwent family interviews and received physical and laboratory examinations at a Mobile Examination Center (MEC) to obtain data on disease status, dietary habits and nutrition, dietary intake, as well as behavioral and socioeconomic characteristics. It should be noted that fasting plasma glucose (FPG) and related fasting biochemical parameters were measured only in those individuals who underwent MEC examinations in the morning and who met the fasting requirement of 8–23 h, denoted as the “fasting subsample.” To attain the national representativeness of this subsample and to reduce the selection bias owing to stratified sampling or non-response, NHANES provides Fasting Subsample MEC Weights (WTSAF4YR and WTSAF2YR). These weights must be used with the particular complex survey design variables (SDMVSTRA and SDMVPSU) in all analyses involving fasting laboratory measures. Those individuals designated for the fasting subsample but who failed to complete fasting blood draws or laboratory tests were assigned a WTSAF2YR value of “0” and were excluded from analyses requiring fasting weights.

**Fig. 1:**
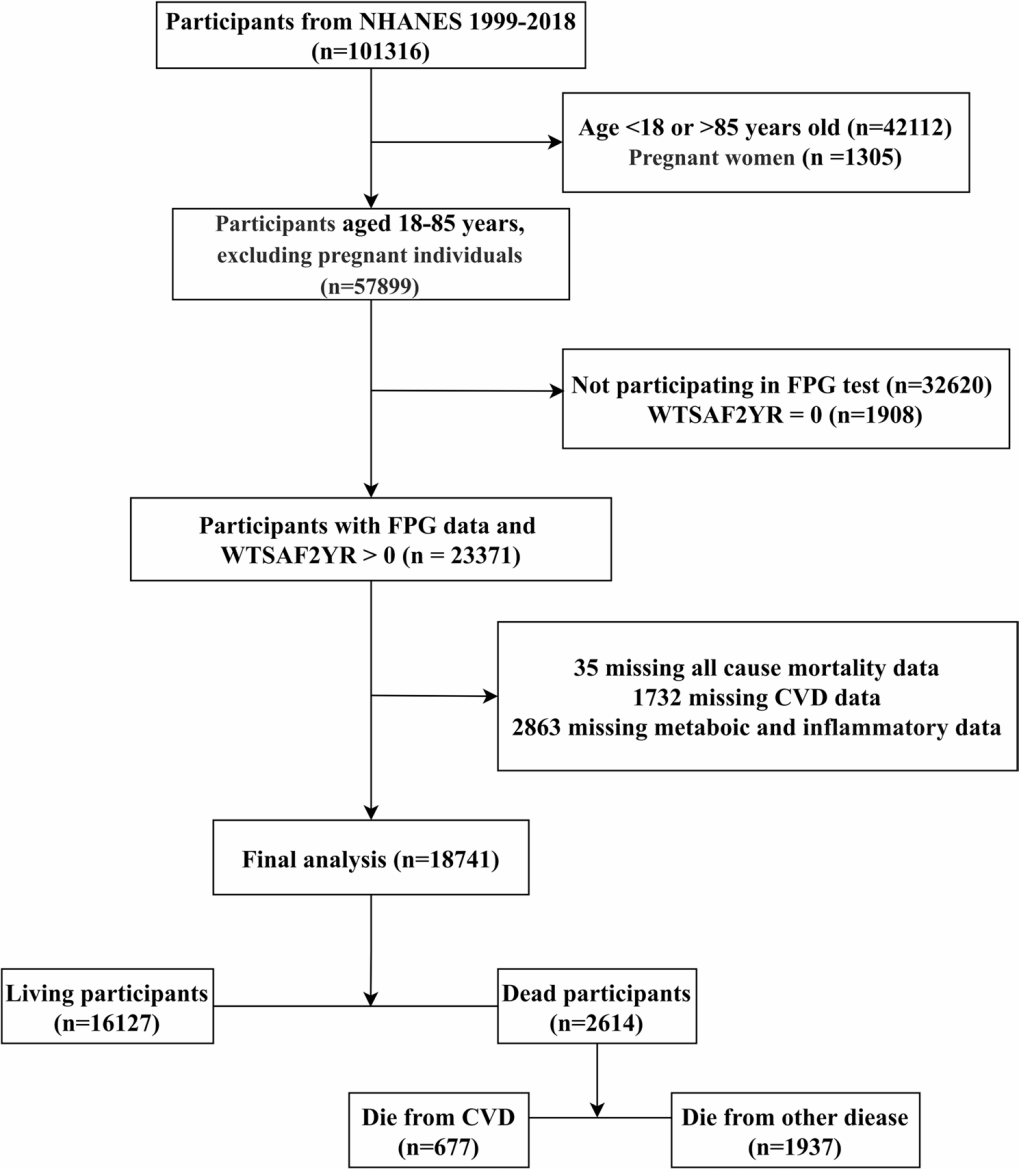
Flow diagram of study participants from NHANES 1999–2018. Abbreviations: NHANES, National Health and Nutrition Examination Survey; CVD, cardiovascular disease

The protocol for participant selection is illustrated in Fig. 1. Initially, participants aged 18–85 years who were not pregnant were included (*n* = 57,899); those aged under 18 years or over 85 years (*n* = 42,112) and pregnant women (*n* = 1,305) were excluded. Subsequently, individuals without FPG measurements or with WTSAF4YR and WTSAF2YR = 0 were excluded (*n* = 34,528), making up a fasting subsample of individuals with valid FPG measurements and positive sample weights (*n* = 23,371). After excluding individuals with missing death information (*n* = 35), those with missing CVD information (*n* = 1,732), and those with partially missing metabolic or inflammatory data (*n* = 2,863), a total of 18,741 participants were ultimately included in the analysis. During follow-up, these participants translated into 16,127 survivors and 2,614 deaths, of which 677 were owing to CVD. The missing data for other variables underwent complete-case analysis on the grounds of a stated methodological rationale; the baseline comparisons between included and excluded participants are presented in Table S1 to assess selection bias.

### Definition of TyG and AIP

In this study, TyG was defined as follows:

$$\:TyG={ln}\left(\frac{fasting\:serum\:TG(mg/dL)\times\:\mathrm{F}\mathrm{P}\mathrm{G}(\mathrm{m}\mathrm{g}/\mathrm{d}\mathrm{L})}{2}\right)$$

where TG refers to triglyceride. Some studies have validated this formula, which was used as a valid surrogate indicator of IR. Enzymatic methods were used to determine TG and blood glucose levels [17].

AIP was defined as follows [16]:

$$\:AIP={{log}}_{10}\left(\frac{fasting\:serum\:TG(mmol/L)}{HDL-C(mmol/L)}\right)$$

where HDL-C refers to high-density lipoprotein cholesterol. In the statistical analysis, the participants were assigned to three categories (T1–T3) based on tertiles of baseline TyG and AIP, with T1 serving as the reference category. This grouping method has been commonly used in related population studies [18, 19].

### Definition of CVD

In NHANES, the history of CVD was assessed using the standardized Medical Conditions questionnaire (MCQ). Individuals were defined as having CVD if they answered yes to any inquiries regarding a previous diagnosis of the following made by a clinician or other healthcare professional: stroke (MCQ160f), myocardial infarction or heart attack (MCQ160e), angina or angina attack (MCQ160d), coronary heart disease (MCQ160c), or congestive heart failure (MCQ160b). The wording of these items is explicitly defined in the CDC/NCHS MCQ codebook and has been consistently applied across NHANES survey cycles.

### Definition of mortality

In NHANES, mortality data were acquired through a probabilistic matching linkage with the NCHS National Death Index (NDI). These outcomes are available in the publicly accessible linked mortality files (LMFs). The follow-up cutoff date for the public LMFs was December 31, 2019. Each participant was monitored from the date of the household interview or MEC examination until either the date of death or the follow-up cutoff date. For participants adjudicated as alive, the follow-up duration was assigned up to December 31, 2019. All-cause mortality was defined as death events with MORTSTAT = 1 in the LMFs, and CVD mortality was identified based on the underlying cause of death recode variable UCOD_LEADING provided in the LMFs.

### Definitions of DII and SIRI

Two 24-h dietary recalls were administered to evaluate dietary intake. The initial recall took place in person and was carried out by trained interviewers who used the Automated Multiple-Pass Method developed by the United States Department of Agriculture at the MECs. The second recall occurred via a telephone call between 3 and 10 days afterward. Based on the data collection, the NHANES “Total Nutrient Intakes” dataset was generated, converting food items into nutrient quantities. DII is a score based on existing research that assesses how much a diet can contribute to inflammation, analyzing both pro-inflammatory and anti-inflammatory aspects related to systemic inflammation. Construction of the DII relies on reference intakes from 11 global dietary databases and a systematic review that integrates over 6,000 research articles [10, 20]. Existing research indicates that using fewer than 30 food components can yield a DII with stable predictive capability [10, 21]. Considering constraints regarding the variety of dietary components examined by NHANES, 27 components were selected for calculation of the DII in this study [21]. These components were vitamins A, B1, B2, B3, B6, B12, C, D, E, folate, carbohydrates, alcohol, fiber, β-carotene, caffeine, protein, total fat, cholesterol, n-3 fatty acids, saturated fatty acids, n-6 fatty acids, polyunsaturated fatty acids, monounsaturated fatty acids, zinc, iron, selenium, and magnesium [21, 22]. The calculation formula for DII is as follows [10]:

$$\:DII=\sum\:_{i=1}^{k}\left({C}_{i}\times\:IE{S}_{i}\right)$$

$$\:{z}_{i}=\frac{{x}_{i}-{u}_{i}}{{\sigma\:}_{i}}$$

$$\:{C}_{i}={z}_{i}\to\:2\times\:\left(transformed\:into\:a\:percentile\:score\right)-1$$

The inflammatory effect score is represented by IES. The daily average intake is denoted by Xi; µi represents the global daily mean intake, and σi represents the standard deviation [21].

Hematological indices were gathered and assessed at MECs using the fully automated Beckman Coulter hematology analyzers. These analyzers assess full blood counts and also five-part differentials for white blood cells by means of volume, conductivity, and scatter technology. Quality control was carried out in accordance with set standards, ensuring compliance with the Clinical Laboratory Improvement Amendments provisions. Monocyte, neutrophil, and lymphocyte counts were obtained from complete blood counts, with results expressed in units of ×10^3^ cells per milliliter. The SIRI was calculated as follows:

$$\:SIRI=\frac{\mathrm{m}\mathrm{o}\mathrm{n}\mathrm{o}\mathrm{c}\mathrm{y}\mathrm{t}\mathrm{e}\:\mathrm{c}\mathrm{o}\mathrm{u}\mathrm{n}\mathrm{t}\:\times\:\:\mathrm{n}\mathrm{e}\mathrm{u}\mathrm{t}\mathrm{r}\mathrm{o}\mathrm{p}\mathrm{h}\mathrm{i}\mathrm{l}\:\mathrm{c}\mathrm{o}\mathrm{u}\mathrm{n}\mathrm{t}}{\mathrm{l}\mathrm{y}\mathrm{m}\mathrm{p}\mathrm{h}\mathrm{o}\mathrm{c}\mathrm{y}\mathrm{t}\mathrm{e}\:\mathrm{c}\mathrm{o}\mathrm{u}\mathrm{n}\mathrm{t}}$$

Values are represented as ×10^3^ cells/mL, in accordance with earlier studies [23, 24]. However, it is important to acknowledge that using ratios as exposure variables in regression models and complex statistical analyses may introduce complications and potentially violate the assumptions underlying causal inference. For statistical analyses, the participants were divided into three groups (T1–T3), one for each tertile of baseline DII and SIRI, with T1 taken as the reference group. This design has been used frequently in relevant population studies [6, 25].

### Covariates

Covariates were selected in accordance with the existing literature [6, 21, 26], and they have been shown to be associated with CVD, inflammation, metabolism, and diet quality. Covariates encompass the following: (1) sociodemographic characteristics: age, sex, race and ethnicity, and family income (expressed as the poverty income ratio [PIR], categorized as poor: PIR < 1 and non-poor: PIR ≥ 1); (2) physiological indicators and medical history: a history of hypertension (or blood pressure measurements ≥ 140/90 mmHg on 3 separate days) and body mass index (BMI); (3) lifestyle habits: smoking habit (defined as having smoked a minimum of 100 cigarettes throughout one’s lifetime), physical activity status (with high-level physical activity classified as ≥ 600 metabolic equivalents (METs)-min/week and light physical activity categorized as ≤ 600 MET-min/week) [27, 28], and average energy intake. It is noteworthy that alcohol intake and history of diabetes (determined according to a self-reported diabetes history, insulin injections, hypoglycemic medication use, glycated hemoglobin [HbA1c] ≥ 6.5%, and FPG ≥ 126 mg/dL) [29] were not adjusted for; these are factored into calculations of the DII and TyG indices.

### Statistical analysis

All analyses accounted for the complex specifications of NHANES’ multi-stage sampling design and inference corrected for design weights, stratification, and clustering. According to NHANES analysis standards, the data for all participants were weighted using the recommended Fasting Subsample MEC Weights (WTSAF4YR and WTSAF2YR). Continuous variables that are normally distributed are presented as the weighted mean and standard deviation, and they were analyzed using analysis of variance. With an interquartile range indicating a skewed distribution, the Kruskal–Wallis test was used; categorical variables represented by weighted percent were assessed using the chi-square test. Spearman correlation analysis was used to assess the relationships among DII, SIRI, TyG, and AIP; weighted linear regression was applied to measure these linear associations.

Logistic regression with design weights was used to study the associations between TyG, AIP, DII, SIRI, and CVD prevalence. The results are presented as odds ratios (ORs) and 95% confidence intervals (CIs). A forest plot was used to visualize the results of the total adjusted model. In Model 1, population and sociodemographic factors such as sex, age, race and ethnicity, and socioeconomic level were adjusted for. In Model 2, further adjustments were made to account for the physiological variables BMI and a history of hypertension. The fully adjusted Model 3 also accounted for lifestyle factors such as smoking habit, physical activity, and average energy intake. Restricted cubic splines (RCS) models were used to examine the relationship of each indicator with the outcomes. Non-linearity was tested with the joint Wald test for the spline terms. Smooth effect curves were plotted, with the inflection point of the curve being where the risk transitioned from “non-significant” to “significant.” The model’s outcome of logistic regression for CVD was assessed with the receiver operating characteristic (ROC) curve; an area under the ROC curve (AUC) > 0.8 indicated high accuracy [30].

The relationships among the above four indicators and all-cause as well as CVD mortality were evaluated using Cox proportional hazards models. The results are presented as hazard ratios (HRs), accompanied by 95% CIs to indicate the precision of these estimates. In a similar manner, forest plots for the fully adjusted model were created, and RCS was used to investigate possible nonlinear associations. Kaplan–Meier survival curves were plotted based on tertile groups of the four indices, and the accuracy was evaluated using ROC curves and the AUC.

To investigate the mediating functions of both the DII and SIRI in the pathway from TyG/AIP to the three outcomes (CVD, along with CVD and all-cause mortality), the percentage of mediation was calculated. Quantification was performed using the ratio of the indirect effect to the total effect, with significance tested through 1,000 bootstrap resamples [31]. This calculation enabled us to assess the degree to which these mediators may contribute to this pathway.

To assess the interaction and joint effects of pairwise combinations of the four study indicators (TyG, AIP, DII, and SIRI) on the three outcomes (CVD prevalence, CVD mortality, and all-cause mortality), simultaneous multiplicative and additive interaction assessment was conducted for these outcomes. The joint effects were reported as ORs or HRs with 95% CIs for each group. In the analysis, T1 + T1 was taken as the reference category. False discovery rate (FDR) correction was then applied for each category of hypothesis test. The three categories of hypothesis tests applied were regression analysis, interaction effect analysis, and joint effect analysis. This was done to control for Type I error rate inflation owing to multiple comparisons. The FDR-corrected *P*-values are reported accordingly. To assess how missing data affected the main outcomes, multiple imputations were performed on the population with missing metabolic/inflammatory data and sensitivity analyses were conducted.

The statistical analysis was carried out using R (version 4.4.1; The R Project for Statistical Computing, Vienna, Austria). The “survey” package was used to conduct weighted logistic and Cox regression analyses. ROC analyses were performed using the “pROC” package, as were mediation effects models with the “mediation” and “CMAverse” packages. A two-sided *P*-value < 0.05 was considered as statistically significant.

## Results

### Population characteristics

The weighted baseline traits of the study population are presented in Table 1. Among the 18,741 U.S. adults included, 2,039 adults were diagnosed with CVD, representing 10.8% of the sample. The weighted prevalence of CVD demonstrated statistically significant differences across various factors, including age, sex, BMI, waist circumference, race and ethnicity, PIR, smoking habit, alcohol intake, average energy intake, physical activity, dietary indicators, and laboratory indicators (all *P* < 0.05), with the exception of lymphocyte counts (*P* = 0.133). Furthermore, a greater prevalence of diabetes and hypertension was found within the CVD cohort (both *P* < 0.001). Notably, the OR [95% CI] values for the SIRI (0.97 [0.68, 1.39] vs. 1.29 [0.87, 1.91]), DII (1.66 [0.12, 2.87] vs. 2.00 [0.57, 3.18]), TyG (8.56 [0.60] vs. 8.82 [0.64]), and AIP (− 0.06 [0.31] vs. 0.03 [0.31]) were all significantly elevated in the CVD group (all *P* < 0.001). Inflammation-related indices (DII and SIRI, as well as blood cell counts) and blood glucose indicators (FPG and HbA1c) showed no significant differences between the excluded (*n* = 4,630) and included participants (all *P* > 0.05). However, the excluded participants exhibited significantly higher metabolic indices (TyG and AIP, as well as lipid profile indices) than those included in the analysis (Table S1).

**Table 1:** Baseline characteristics according to the CVD status

Characteristics	CVD status		*P* value
	Non-CVD (*N* = 16702)	CVD (*N* = 2039)	
General characteristics			
**Gender (%)**			< 0.001
Male	8040 (47.9)	1168 (55.5)	
Female	8662 (52.1)	871 (44.5)	
**Age**,** y**,** mean (SD)**	45.29 (16.07)	63.87 (13.21)	< 0.001
**Race (%)**			< 0.001
Mexican American	3077 (8.5)	222 (4.0)	
Non-Hispanic Black	3235 (10.7)	413 (11.2)	
Non-Hispanic White	7368 (68.6)	1154 (75.3)	
Other	3022 (12.1)	250 (9.5)	
**Marital (%)**			0.897
Yes	10,328 (65.0)	1203 (65.2)	
No	6374 (35.0)	836 (34.8)	
**Income-to-poverty (%)**			0.005
Not poor	13,775 (87.8)	1641 (85.1)	
Poor	2927 (12.2)	398 (14.9)	
**Education status (%)**			< 0.001
Below high school	4114 (15.9)	699 (26.0)	
High School or above	12,588 (84.1)	1340 (74.0)	
**BMI**,** kg/m**^**2**^, **mean (SD)**	28.62 (6.64)	30.03 (6.86)	< 0.001
**Waist circumference**,** cm**,** mean (SD)**	97.78 (16.25)	105.11 (16.23)	< 0.001
**Abdominal obesity (%)**			< 0.001
Yes	12,352 (72.6)	1810 (87.8)	
No	4350 (27.4)	229 (12.2)	
**Average energy intake**,** kcal**,** mean (SD)**	0.00 (0.15)	−0.04 (0.14)	< 0.001
**Smoke (%)**			< 0.001
Yes	7309 (44.7)	1270 (62.3)	
No	9393 (55.3)	769 (37.7)	
**Alcohol (%)**			< 0.001
Yes	12,343 (73.9)	1309 (64.6)	
No	1982 (11.9)	246 (35.4)	
Missing	2377 (14.2)	484 (23.7)	
**Physical activity (%)**			< 0.001
Light physical activity	8216 (46.3)	1221 (55.7)	
High level of physical activity	8486 (53.7)	818 (44.3)	
**Diabetes (%)**			< 0.001
Yes	2362 (14.1)	765 (37.5)	
No	14,340 (85.9)	1274 (62.5)	
**Hypertension (%)**			< 0.001
Yes	6289 (10.5)	1576 (33.5)	
No	10,413 (89.5)	463 (66.5)	
**Laboratory characteristics**			
** Neutrophils number**,** mean (SD)**,** 10**^**9**^**/L**	3.95 (1.59)	4.36 (1.65)	< 0.001
** Lymphocyte number**,** mean (SD)**,** 10**^**9**^**/L**	2.01 (0.90)	1.96 (1.87)	0.133
** Monocyte number**,** mean (SD)**,** 10**^**9**^**/L**	0.53 (0.19)	0.60 (0.22)	< 0.001
** HbA1c**,** %**,** mean (SD)**	5.52 (0.83)	6.04 (1.16)	< 0.001
** FPG**,** mmol/L**,** mean (SD)**	5.73 (1.45)	6.50 (2.15)	< 0.001
** LDL-cholesterol**,** mmol/L**,** mean (SD)**	3.02 (0.89)	2.71 (1.00)	< 0.001
** HDL-cholesterol**,** mmol/L**,** mean (SD)**	1.40 (0.41)	1.33 (0.42)	< 0.001
** TC**,** mmol/L**,** mean (SD)**	5.04 (1.01)	4.75 (1.15)	< 0.001
** TG**,** mmol/L**,** mean (SD)**	1.34 (0.75)	1.55 (0.81)	< 0.001
** Uric acid**,** umol/L**,** mean (SD)**	322.05 (80.89)	352.59 (93.21)	< 0.001
**Inflammation-related indices**			
SIRI, mean (SD)	0.97 (0.68, 1.39)	1.29 (0.87, 1.91)	< 0.001
DII, mean (SD)	1.66 (0.12, 2.87)	2.00 (0.57, 3.18)	< 0.001
**Metabolic indicators**			
TyG, mean (SD)	8.56 (0.60)	8.82 (0.64)	< 0.001
AIP, mean (SD)	−0.06 (0.31)	0.03 (0.31)	< 0.001

Note: Continuous variable with a normal or skewed distribution was presented as mean (SD) or median (25th-75th percentile), and categorical variables were presented as numbers (percentages). Variables between groups were compared by variance test, Kruskal-Wallis test, or Chi-square test

*CVD* Cardiovascular Disease, *BMI* body mass index, *HbA1c* Hemoglobin A1c, *FPG* Fasting Plasma Glucose, *LDL* Low-Density Lipoprotein, *HDL* High-Density Lipoprotein, *TC* Total Cholesterol, *TG* Triglyceride, *SIRI* System Inflammation Response Index, *DII* Dietary Inflammatory Index, *TyG* Triglyceride Glucose Index, *AIP* Atherogenic index of plasma, *SD* standard deviation

### Correlations between inflammation-related indices and metabolism indicators

The correlation analysis using the Spearman correlation matrix revealed multiple significant correlations between metabolic indicators and inflammation-related indicators across both the full sample and stratified groups (non-CVD and CVD), demonstrating consistent overall directions (Fig. S1). The results of linear regression demonstrated that DII showed a consistent positive relationship with metabolic indicators (TyG and AIP). Specifically, in Model 3, DII was significantly positively associated with TyG (β = 0.12, 95% CI 0.07–0.18, *P* < 0.001) and AIP (β = 0.33, 95% CI 0.22–0.43, *P* < 0.001), showing slightly attenuated effect sizes compared with the crude model while maintaining consistent directions (Table S2). However, the association between the SIRI and metabolic indicators was significant in the unadjusted model, but it became non-significant after adjusting for covariates such as sociodemographics, physiological indicators, medical history, and lifestyle habits. In Model 3, the associations of SIRI with TyG (β=−0.03, *P* = 0.080) and AIP (β=−0.04, *P* = 0.200) did not achieve statistical significance (Table S3). To further explore the associations between metabolic indices (TyG and AIP) and systemic inflammation, the linear relationships between SIRI components (neutrophil, monocyte, and lymphocyte counts) and TyG and AIP were examined (Table S4). In the fully adjusted model, the neutrophil count was significantly associated with TyG (β = 0.32, 95% CI 0.27–0.38, *P* < 0.001). Similarly, the monocyte count was significantly associated with both the TyG (β = 0.01, 95% CI 0.01–0.02, *P* < 0.001) and AIP (β = 0.04, 95% CI 0.03–0.05, *P* < 0.001). Additionally, the lymphocyte count was significantly associated with TyG (β = 0.22, 95% CI 0.19–0.25, *P* < 0.001) and AIP (β = 0.46, 95% CI 0.40–0.52, *P* < 0.001).

### Associations of metabolism indicators and inflammation-related indices with CVD prevalence and mortality

**Fig. 2:**
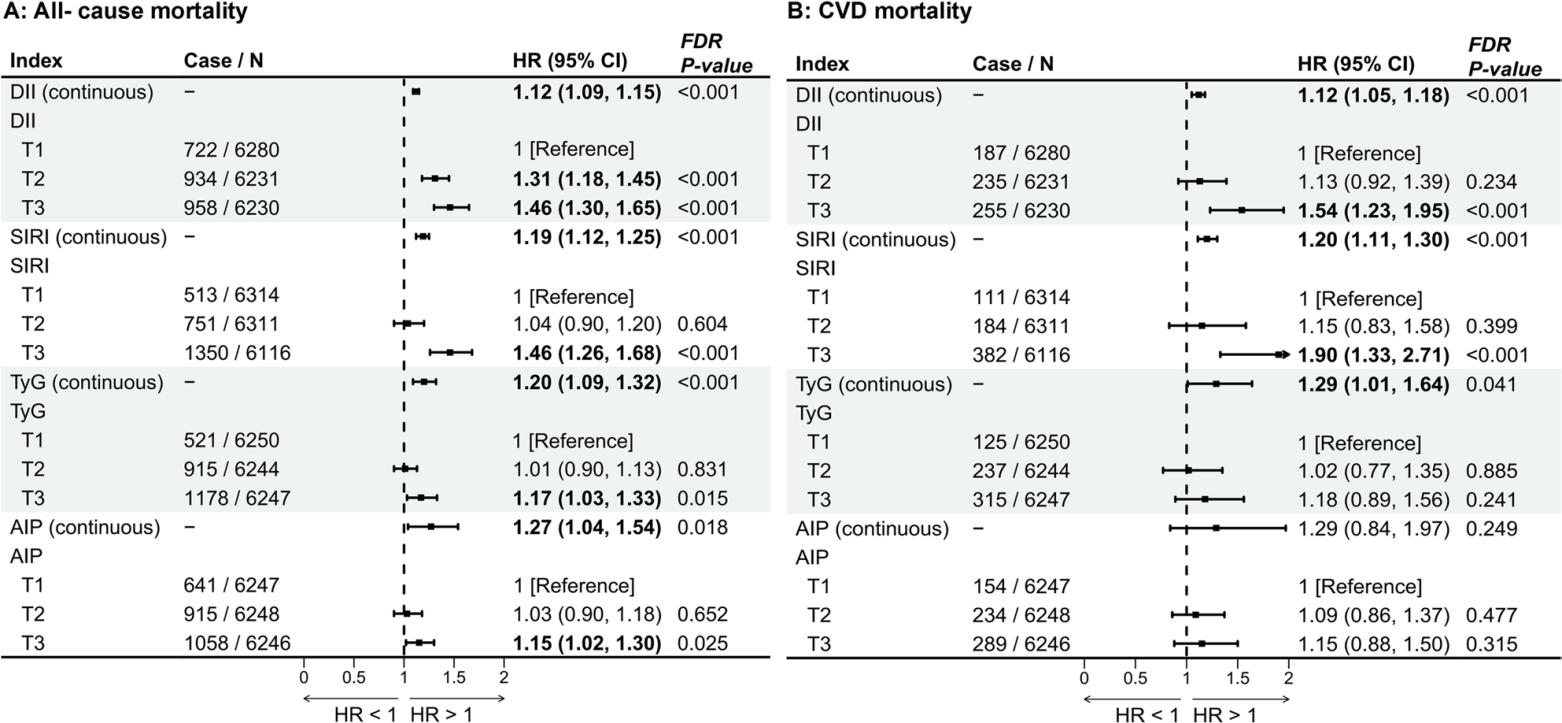
Associations of inflammation-related indices and metabolic indicators with mortality. The model was adjusted for sex, age, race and ethnicity, socioeconomic level, BMI, hypertension, smoking habit, energy intake, and exercise status. The figure shows *P*-values corrected by the FDR. (**A**) all-cause mortality; (**B**) CVD mortality. Abbreviations: CVD, cardiovascular disease; DII, dietary inflammatory index; SIRI, systemic inflammatory response index; TyG, triglyceride–glucose index; AIP, atherogenic index of plasma; BMI, body mass index; HR, hazard ratio; CI, confidence interval; T, tertile; FDR, false discovery rate. HRs showing statistical significance (*P* < 0.05) are presented in bold

A total of 2,039 patients with CVD were included. During an average follow-up duration of 10.02 years, 2,614 deaths were recorded, of which 677 were attributed to CVD (Fig. 1; Table 1). Forest plots from the fully adjusted model (Model 3), which controlled for age, sex, race and ethnicity, BMI, smoking status, energy intake, physical activity, and history of hypertension, are presented in Fig. 2 and S2. These showed that both metabolic indices (TyG and AIP) and inflammation-related indices (DII and SIRI) were significantly associated with an increased prevalence of CVD and risk of mortality, with all FDR-adjusted *P*-values remaining statistically significant. Compared with the lowest tertile, the highest tertiles of AIP, DII, and SIRI were significantly associated with higher CVD prevalence [OR (95% CI): 1.40 (1.17–1.67) for AIP; 1.30 (1.06–1.61) for DII; and 1.50 (1.25–1.80) for SIRI]. The highest tertile of TyG tended to be associated with an increased CVD prevalence. In terms of mortality outcomes, Cox proportional hazards analyses indicated that, relative to the lowest tertile, higher tertiles of TyG, AIP, DII, and SIRI were significantly associated with increased risks of all-cause mortality—by 17% (95% CI: 3%–33%), 15% (95% CI: 2%–30%), 46% (95% CI: 30%–65%), and 46% (95% CI: 26%–68%), respectively. Furthermore, participants in the highest tertile of DII and SIRI had 54% and 90% higher risks of CVD mortality, respectively, compared with those in the lowest tertile [HR (95% CI): 1.54 (1.23–1.95) for DII; 1.90 (1.33–2.71) for SIRI]. By contrast, the associations of TyG and AIP with CVD mortality were not statistically significant [HR (95% CI): 1.18 (0.89, 1.56), *P* = 0.241 for TyG; 1.15 (0.88, 1.50), *P* = 0.315 for AIP] **(**Fig. 2**)**. Tables S5 and S6 present the continuous and categorical results for Models 1 and 2, respectively, showing general consistency with the findings from Model 3.

**Fig. 3:**
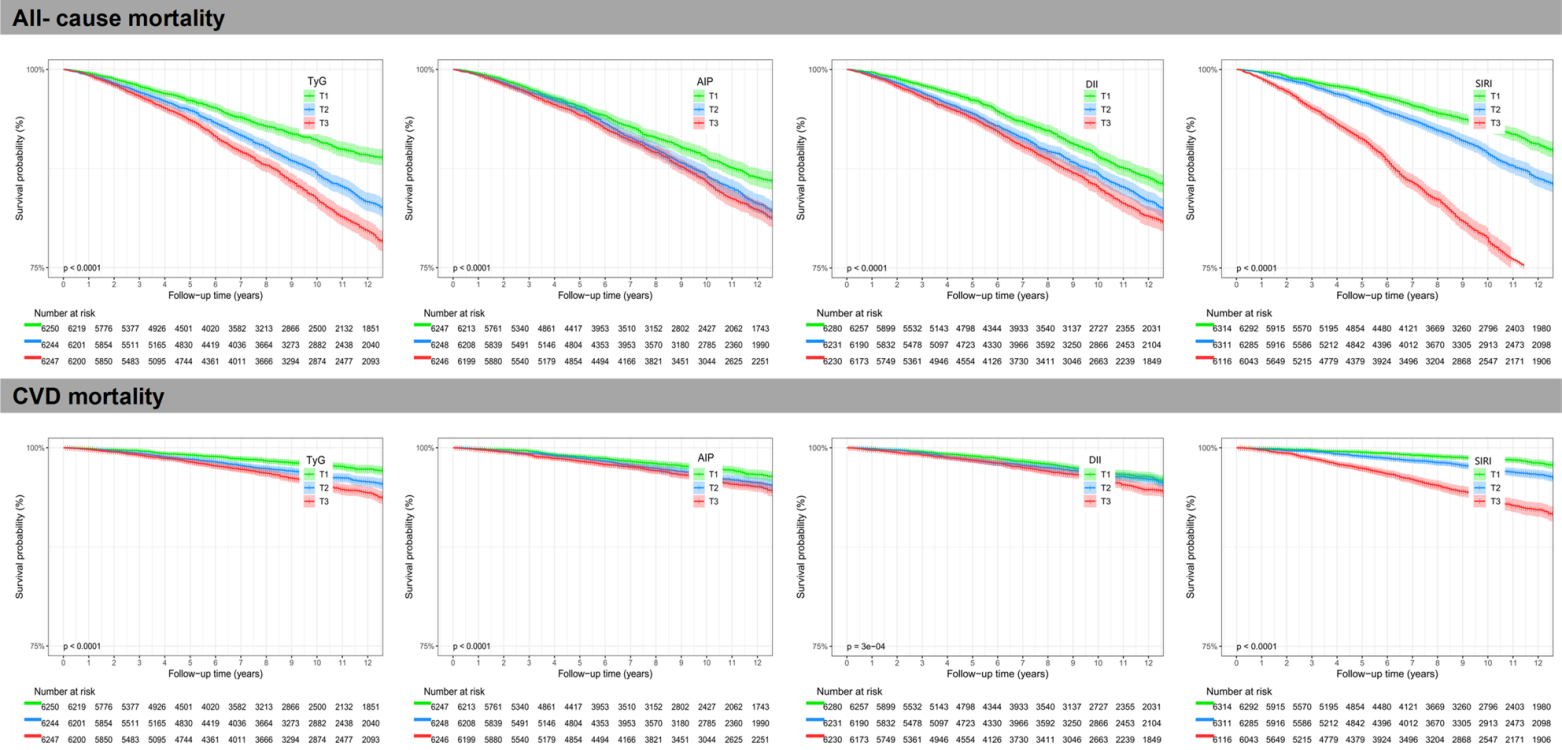
Kaplan–Meier survival curves according to inflammation-related indices (DII and SIRI) and metabolic indicators (TyG and AIP). Abbreviations: CVD, cardiovascular disease; DII, dietary inflammatory index; SIRI, systemic inflammatory response index; TyG, triglyceride–glucose index; AIP, atherogenic index of plasma; T, tertile

Kaplan–Meier survival curves, divided by tertiles of the inflammation-related indices and metabolic indicators (illustrated in Fig. 3), indicated poorer survival outcomes in patients with higher tertiles of these indices. By contrast, groups in the lower tertiles exhibited improved overall survival rates.

**Fig. 4:**
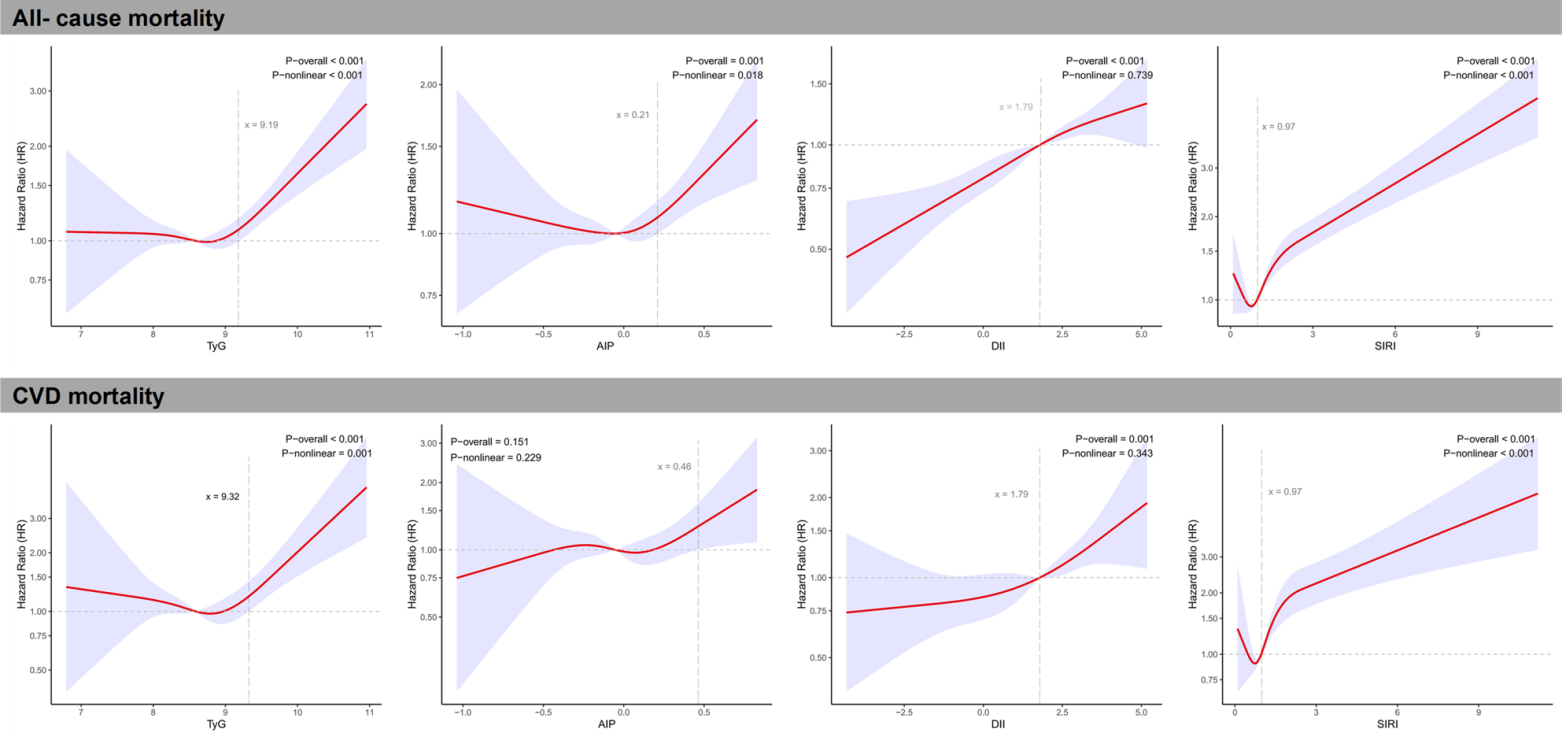
Generalized additive model (GAM) illustrating the association of metabolic indicators and inflammation-related indices with mortality. The model was adjusted by sex, age, race and ethnicity, socioeconomic level, BMI, hypertension, smoking habit, energy intake, and exercise status. The vertical dotted line is the inflection point where the risk changes from “nonsignificant” to “significant.” Abbreviations: CVD, cardiovascular disease; DII, dietary inflammatory index; SIRI, systemic inflammatory response index; TyG, triglyceride–glucose index; AIP, atherogenic index of plasma; BMI, body mass index; HR, hazard ratio; CI, confidence interval

Based on logistic and Cox regression analyses, it is proposed that non-linear relationships may exist between the continuous TyG, AIP, DII, and SIRI indices and CVD as well as mortality outcomes. Figure 4 and S3 illustrate smooth curves depicting the relationships among these four indices and the results obtained from a generalized additive model. Both metabolic indicators (TyG and AIP) demonstrated U-shaped associations with CVD prevalence (Fig. 4) and J-shaped associations with all-cause mortality (Fig. 4). Specifically, TyG exhibited a J-shaped relationship with CVD mortality (Fig. 4); the RCS curve results for AIP and CVD mortality were aligned with the Cox regression model, revealing no significant differences (*P* = 0.151). However, the DII and SIRI exhibited a consistent upward trend related to the three adverse cardiovascular outcomes (Fig. 4 and S3). To enhance the clinical applicability of this study, the x-axis values corresponding to the intersections of all RCS curves with OR/HR = 1 were also calculated, as presented in Fig. 4 and S3. The assessment of models was performed using ROC curves. When the four indices were analyzed as continuous variables, AUCs for all fully adjusted logistic and Cox regression models surpassed 0.80 (Fig. S4). Additionally, in the crude models evaluating the predictive power of the four indices for the three outcomes (Fig. S5), SIRI demonstrated the highest predictive value (AUC = 0.615 for CVD prevalence, 0.628 for all-cause mortality, and 0.642 for CVD mortality), followed by TyG (AUC = 0.596 for CVD prevalence, 0.603 for all-cause mortality, and 0.606 for CVD mortality), all with *P*-values < 0.001.

### Mediation effects of DII and SIRI on TyG– and AIP–CVD and mortality associations

**Fig. 5:**
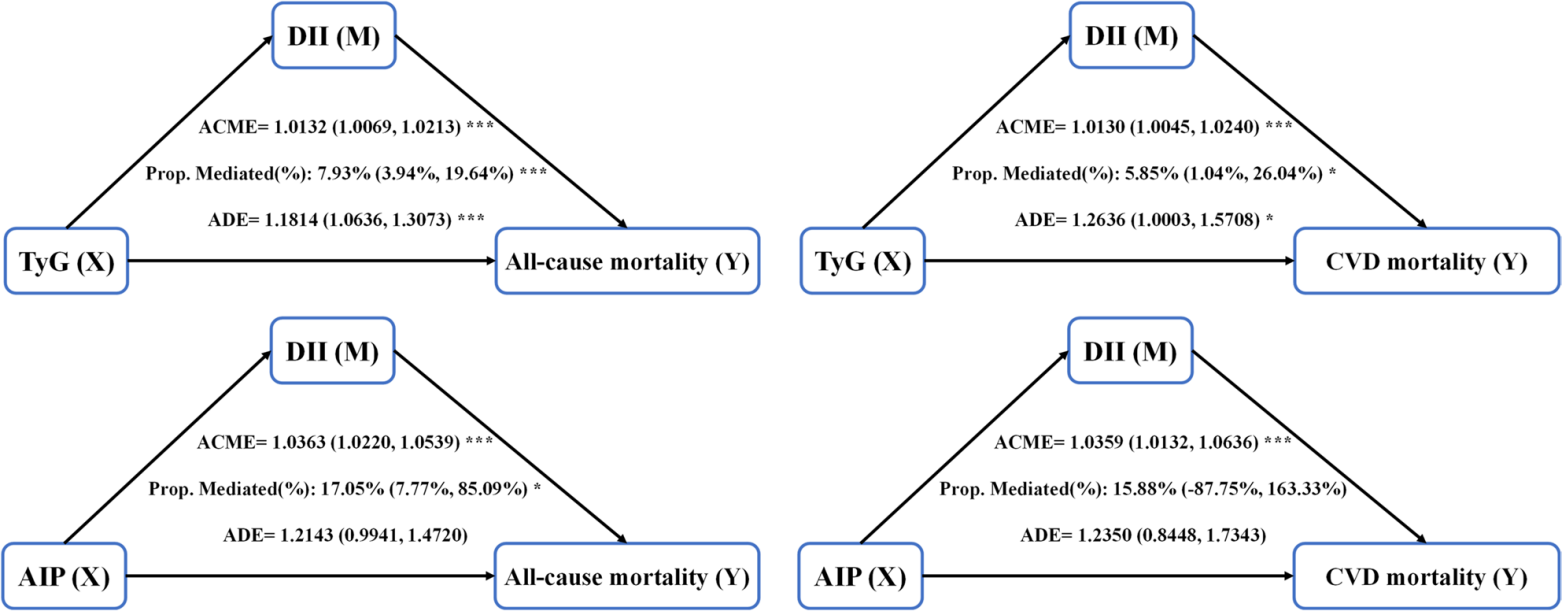
Mediation effects of DII in the associations of metabolic indicators with all-cause and CVD mortality. The model was adjusted by sex, age, race and ethnicity, socioeconomic level, BMI, hypertension, smoking habit, energy intake, and exercise status. Abbreviations: ACME, average causal mediation effects (indirect effect); ADE, average direct effects; DII, dietary inflammatory index; TyG, triglyceride–glucose index; AIP, atherogenic index of plasma. * *P* < 0.05, ** *P* < 0.01, and *** *P* < 0.001

Figure 5 and Table S7 present the mediation effects of DII and SIRI on the associations between metabolic indicators (TyG and AIP) and the three outcomes (CVD prevalence, all-cause mortality, and CVD mortality). DII demonstrated a positive partial mediating effect on the relationships involving both metabolic markers (TyG and AIP) and a higher CVD prevalence, as well as a greater risk of all-cause and CVD mortality. In terms of analyzing CVD prevalence, the TyG pathway accounted for 3.58% (95% CI 3.50%–3.66%), and the AIP pathway contributed 3.99% (95% CI 3.93%–4.07%) (Table S7). In terms of analyzing all-cause mortality, the TyG pathway accounted for 7.93% (95% CI 3.94%–19.64%), and the AIP pathway accounted for 17.05% (95% CI 7.77%–85.09%) (Fig. 5). In the analysis of CVD mortality, the TyG pathway accounted for 5.85% (95% CI 1.04%–26.04%), whereas the AIP pathway did not yield statistically significant results: 15.88% (95% CI − 87.75%, 163.33%) (Fig. 5). Furthermore, in contrast to DII, SIRI exhibited a negative mediating effect. In the analysis of CVD prevalence, the pathway proportions for TyG and AIP were − 2.09% (95% CI − 2.14%, − 2.05%) and − 1.25% (95% CI − 1.28%, − 1.21%), respectively (Table S7). In the analysis of all-cause mortality, the pathway proportion for TyG was − 2.84% (95% CI − 10.36%, − 0.03%); the AIP pathway did not demonstrate statistical significance: −3.05% (95% CI − 17.00%, 2.11%) (Table S7). In the analysis of CVD mortality, neither the pathway proportions for TyG (− 2.34% [95% CI − 12.98%, 0.51%]) nor those for AIP (− 3.02% [95% CI − 31.67%, 23.23%]) reached statistical significance (Table S7).

Given the negative mediating role of SIRI in the associations between metabolic indices (TyG and AIP) and adverse cardiovascular outcomes, the mediating effects of SIRI components (neutrophil, monocyte, and lymphocyte counts) on these associations were further explored (Table S8). The results showed that neutrophil and monocyte counts exerted positive mediating effects on the relationships between metabolic indices (TyG and AIP) and adverse cardiovascular outcomes. By contrast, lymphocyte count exhibited a negative mediating effect in the association between metabolic indices and cardiovascular mortality while showing positive mediating effects in the associations with CVD prevalence and all-cause mortality.

### Interaction and joint analysis of inflammation-related indices and metabolism indicators with CVD prevalence and mortality

After adjustment for age, sex, race and ethnicity, BMI, smoking status, energy intake, physical activity, and history of hypertension, the AIP and DII indices demonstrated a statistically significant positive additive interaction on the risk of CVD, with a relative excess risk due to interaction of 0.49 (95% CI 0.07–0.91, *P* = 0.022) (Fig. S6). However, this interaction did not remain statistically significant after FDR correction (FDR-*P* = 0.12). By contrast, the SIRI and TyG indices exhibited a negative multiplicative interaction concerning CVD mortality outcomes, with an HR for interaction of 0.61 (95% CI 0.42–0.89, *P* = 0.035) (Fig. S7), which also did not remain statistically significant following FDR adjustment (FDR-*P* = 0.079).

**Fig. 6:**
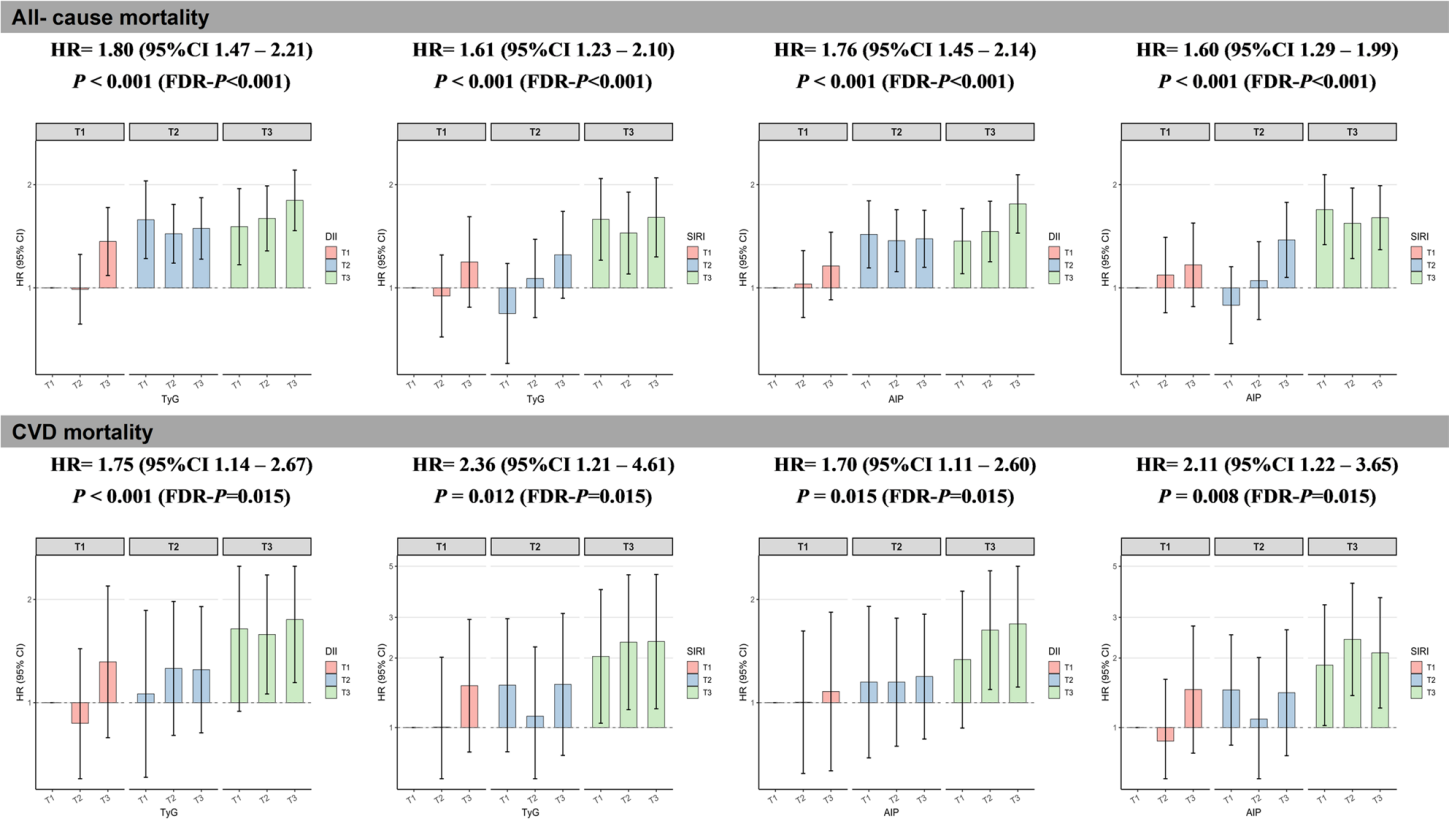
Joint associations of metabolic indicators (TyG and AIP) and inflammation-related indices (DII and SIRI) with mortality. Hazard ratios were adjusted for sex, age, race and ethnicity, socioeconomic level, BMI, hypertension, smoking habit, energy intake, and exercise status. Abbreviations: CVD, cardiovascular disease; DII, dietary inflammatory index; SIRI, systemic inflammatory response index; TyG, triglyceride–glucose index; AIP, atherogenic index of plasma; BMI, body mass index; HR, hazard ratio; CI, confidence interval; T, tertile

The joint associations of pairwise combinations of metabolic and inflammatory indices with the primary outcomes were then further examined. Figure 6 and S8 illustrate that, in comparison with the reference subgroup (metabolic indicator T1 + inflammation-related indicator T1), the high tertile group (metabolic indicator T3 + inflammation-related indicator T3) exhibited a higher prevalence of CVD (e.g., TyG + DII: OR 1.45 [95% CI 1.02, 2.06] for CVD prevalence). Additionally, this group demonstrated significantly increased risks of all-cause mortality and cardiovascular mortality (e.g., TyG + DII: HR [95% CI] 1.80 [1.47, 2.21] for all-cause mortality and 1.75 [1.14, 2.67] for CVD mortality). All these associations remained statistically significant following FDR correction, suggesting that metabolic indices (TyG and AIP) and inflammation-related indices (DII and SIRI) have significant combined effects on the risks of CVD, all-cause mortality, and cardiovascular mortality.

Assessment in sensitivity analyses using the multiply imputed datasets did not meaningfully change the statistical inference regarding the main outcomes, thereby adding further support to the strength of the findings (Tables S9–S11).

## Discussion

This study adds new knowledge by characterizing the relationship between simultaneous exposure to metabolic indicators and inflammation-related indicators and the prevalence of CVD and risk of mortality. Furthermore, the potential role of inflammation-related indices in the relationship between metabolic indicators and adverse cardiovascular outcomes was explored. The findings suggested that both metabolic indicators (TyG and AIP) and inflammation-related indices (DII and SIRI) are significantly linked to CVD morbidity and mortality. Notably, only a limited fraction of the relationship between metabolic indicators (TyG and AIP) and adverse cardiovascular outcomes was found to be mediated by DII. Therefore, joint effect analysis was further conducted. This analysis revealed that adults with both high metabolic and high inflammation-related indicators showed the greatest prevalence of CVD and risk of mortality.

Significant associations between the metabolic index TyG and a higher prevalence of CVD, increased all-cause mortality, and CVD mortality were identified in the current research. The association of metabolic disorders with the prevalence and mortality of CVDs has been extensively discussed in previous studies. As a well-established alternative marker for IR, TyG has been demonstrated in numerous studies to effectively reflect decreased sensitivity of the body to insulin [32, 33]. In agreement with the main findings of the present study, previous studies conducted across various countries and age groups have reported associations between TyG and a greater incidence of CVD, as well as increased risk of all-cause and CVD mortality [6, 34]. Further, the present study enhances these findings by emphasizing the critical role of inflammation in this relationship. Basic research has shown that IR can impair endothelial nitric oxide (NO) signaling while ensuring relative preservation of the vasoconstrictive endothelin-1 pathway, thereby leading to endothelial dysfunction and atherosclerosis and ultimately increasing the risk of adverse cardiovascular events. The positive association observed in this study provides population-level support for this pathway [35, 36].

Given that dyslipidemia and IR frequently coexist and contribute to CVD and adverse cardiovascular outcomes [37], the association of AIP, a predictor of atherosclerosis and cardiovascular risk, with cardiovascular adverse events was investigated. The findings indicated that AIP was similarly correlated with a heightened likelihood of CVD prevalence and all-cause mortality. This conclusion is supported by cohort studies involving populations with cardiovascular–kidney–metabolic syndrome, adults with diabetes or prediabetes, and older patients with diabetes in intensive care units [38–40]. Notably, AIP is negatively correlated with low-density lipoprotein (LDL) cholesterol particle size [16], indicating that elevated AIP is more likely linked to small dense LDL (sd LDL). The sd LDL phenotype is more readily able to penetrate and accumulate in the arterial wall and is more susceptible to oxidation [41], thereby exhibiting a greater potential to cause atherogenesis [42, 43]. In this study, elevated AIP was observed to be associated with a higher prevalence of CVD and an increased risk of mortality, which is consistent with the aforementioned mechanism.

In exploring the potential mechanisms underlying metabolic abnormalities and adverse cardiovascular outcomes, inflammation was found to mediate the association between metabolism and adverse cardiovascular outcomes. Mediation analysis revealed a positive partial mediation effect of DII on the association of TyG/AIP with CVD and mortality. DII scores are derived from the influence of nutritional effects on pivotal inflammatory markers, including tumor necrosis factor-alpha (TNF-α) and interleukins (IL-6 and IL-1β) [10]. The negative DII score reflects the anti-inflammatory properties of nutritional patterns, and the normal score indicates that nutritional patterns promote inflammation [10]. Moreover, higher DII scores are associated with an increased prevalence of CVD and risk of mortality. Concurrently, a pro-inflammatory diet, marked by greater consumption of inflammatory food ingredients, particularly saturated fats, significantly accelerates telomere shortening [44], a process associated with an increased risk of mortality from various causes [45, 46]. The analyses using linear regression models revealed a notable positive relationship among TyG, AIP, and DII; whereas mediation analysis provided additional insight into the function of DII within the relationship between metabolism and adverse cardiovascular outcomes. From a mechanistic perspective, inflammation serves as a common pathway for adverse cardiovascular outcomes resulting from various metabolic abnormalities. Metabolic abnormalities activate the Toll-like receptor 4–kappa B kinase beta–nuclear factor kappa B pathway [47–49], triggering inflammatory transcription that further induces endothelial dysfunction and the development of atherosclerosis [50], thereby increasing the likelihood of adverse cardiovascular events. However, some of the mediation effects observed exhibited extremely wide or even unreasonable CIs (e.g., AIP → CVD mortality: 15.88% [95% CI − 87.75%, 163.33%]). This indicates instability of the results, which may be attributed to the “ratio instability” that occurs when the total effect (AIP → CVD mortality) is not significant. In such cases, calculating the proportion mediated on the HR scale may result in a denominator close to zero, leading to excessively wide CIs, or even values below 0% or above 100%.

Negative mediating and interaction effects of SIRI were observed, which are inconsistent with the predominant direction of systemic inflammation’s positive mediation between metabolism and outcomes reported by Huang et al. [6] and Xu et al. [51] and also contrary to expectations. A possible explanation for this discrepancy lies in the opposing directions of the indirect effect (average causal mediation effect, ACME) and direct effect (average direct effect) observed in mediation analysis. In the context of SIRI-mediated effects, a negative ACME was consistently identified, which can be attributed to the negative linear correlation between metabolic indices (TyG and AIP) and SIRI. Notably, SIRI had a significant positive correlation with adverse cardiovascular events (see Table S3; Fig. 2 and S2). Mooldijk et al. suggested that using ratios as exposure variables results in a violation of the important causal inference criteria, such as the consistency assumption [52]. To further investigate the true relationship between metabolic abnormalities and systemic inflammation, sensitivity analysis was performed to assess components of the SIRI, namely, neutrophil, monocyte, and lymphocyte counts. The metabolic indices (TyG and AIP) were found to be linearly related in a positive manner to neutrophil, monocyte, and lymphocyte counts (Table S4). Furthermore, these cellular counts positively mediated the associations between metabolic indices and both CVD prevalence and all-cause mortality (Table S8). These findings align with expectations and biological plausibility, suggesting that the negative mediation effect of SIRI may stem from spurious correlations or direction reversal caused by shared denominators, mathematical coupling, or amplification of errors by denominators.

Although inflammation has a mediating role between metabolic abnormalities and adverse cardiovascular events, observations indicated that DII accounted for less than 20% of this effect, which suggested that relying solely on an anti-inflammatory diet [53, 54] may not significantly mitigate the risk of diet-related metabolic disorders; thus, other measures are still required to control and manage metabolic abnormalities. Furthermore, inflammation can independently activate the RAAS and the neutrophil and monocyte–macrophage systems, accelerating apoptosis, inducing endothelial damage, and promoting atherosclerosis [9, 55–57]. In the weighted logistic and Cox regression models used herein, inflammatory markers such as DII and SIRI showed a significant association with an increased prevalence of CVD and risk of mortality, aligning with the findings of previous studies [12, 13]. Notably, results showed that the association of these markers with mortality was stronger than that of metabolic indicators. This suggests that inflammation is both a mediator of the association of metabolic abnormalities with adverse cardiovascular events and a risk factor that may independently affect the occurrence and progression of cardiovascular complications [58]. To address this, joint analysis of the interaction and joint effects of metabolic and inflammation-related indicators was performed, measuring their additive or synergistic risk from exposure to different combinations. This approach may provide a solid basis for preventive and therapeutic interventions.

Significant joint effects of the metabolic indices (TyG and AIP) and inflammation-related indices (DII and SIRI) were observed on the prevalence of CVD, all-cause mortality, and CVD mortality. Metabolism may cause inflammation, but inflammation can also result in metabolic disorders. Basic research has shown that TNF-α, secreted by macrophages, can cause IR in adipocytes [59]. Obesity is also related to higher concentrations of inflammatory mediators in adipose tissue, which cause disturbances in glucose metabolism [60, 61]. This highlights the interactions that take place between inflammation and metabolism such that they amplify and interrelate with each other and give rise to a complex immune–metabolic network [62, 63]. In statistical terms, the conjunction of “metabolic indicators × inflammation-related indicators” was found to reflect this. In agreement with these conclusions, Tang et al. [64] showed that the composite indicator of inflammation and IR, the Composite Tumor Index, was significantly correlated with mortality. This study emphasizes that if metabolic disorders (TyG, AIP) and the burden of inflammation (DII, SIRI) are both at higher percentile levels, the prevalence of CVD and the mortality risk will increase significantly. This points to the need to improve lifestyle factors simultaneously with respect to controlling metabolic disorders and modifying dietary patterns to control inflammation [65]. This approach would enhance the accuracy and efficacy of preventive strategies and clinical management of CVD.

This study presents a functional approach for the prevention of CVD. First, the joint control of metabolic risk and lifestyle factors is emphasized. Management of these variables, specifically lipid levels, blood pressure, and blood glucose levels, as well as prescription of an anti-inflammatory diet (such as the Mediterranean and DASH diets), as a part of a combined management strategy may ameliorate dyslipidemia, IR, and the burden of chronic inflammatory disease. Second, clinicians can use the four indices in the present study to perform risk stratification, thereby identifying patients at higher risk for CVD or mortality. Further, the RCS curves indicated that the threshold values are points at which the risk for disease and mortality change from “non-significant” to “significant,” which is also a basis for risk stratification. These points of inflection, however, are not to be taken as clinical cutoff points as they may change in different populations and with different testing procedures. There is an urgent need for external validation and prospective studies examining the clinical applicability of these indices and cutoffs. Based on the present observational mediation results, it is suggested that combining lifestyle and metabolic risk management with dietary management of inflammation may aid in reducing the probability of CVD morbidity and mortality risk. However, these recommendations call for additional evidence, such as from interventional studies, to prove the value of such comprehensive preventive measures.

### Strengths and limitations

The main strength of this research is that this study was based on a nationally representative sample and that it incorporated metabolic indices (TyG, AIP) and inflammation-related indices (DII, SIRI) into a single analytic framework, for the first time, to systematically evaluate the relationship of these indices with the prevalence and mortality of CVD. The research hypothesis was supported by applying a gradual research strategy including mediation analysis, interaction studies, and investigation of joint effects. The results suggested that inflammatory indices act as mediators between metabolism and adverse outcomes in cardiovascular health while reflecting the joint effects of metabolic pathways with inflammatory pathways. This approach fills a large evidence gap regarding these important indices.

Several limitations of this study must also be acknowledged. First, the NHANES-based prevalence analysis used a cross-sectional design. The findings on CVD prevalence are inherently hypothesis-generating, and reverse causality cannot be ruled out (i.e., pre-existing CVD may alter metabolic and inflammatory biomarkers). Furthermore, the mortality analysis, which relied on retrospective linkage with NDI, may be subject to residual confounding and reverse causation. In this study, the prevalence of CVD was derived from self- or proxy-reported responses in the NHANES MCQ, based on the item “Has a doctor or other healthcare professional ever told you…?” to exclude self-diagnosed cases. This approach helps reduce false positives owing to arbitrary self-diagnosis but may still be affected by recall bias and misclassification. Recall bias more commonly manifests as low sensitivity (underreporting) with relatively high specificity, which could lead to an overall underestimation of prevalence and attenuation of exposure–outcome associations. However, if the probability of diagnosis and recall differs across exposure levels (e.g., individuals with greater metabolic risk visit physicians more frequently and are therefore more likely to recall a diagnosis), differential misclassification may occur, resulting in systematic bias with an unpredictable direction. Although participants who were excluded owing to missing covariates did not differ significantly from those included in terms of inflammatory levels, their metabolic levels were significantly higher. This may have led to underestimation of the exposure–outcome associations. Moreover, the lack of evaluation of longitudinal variations in these indices limits the understanding of dynamic effects over time. Also, the follow-up period was relatively short (average of 10.02 years), and individuals who died during the study period may initially have had obvious comorbidities. Some of the mediation effects exhibited very wide CIs, indicating imprecise estimates and necessitating cautious interpretation of these findings. Finally, although major covariates were taken into account, the inherent residual confounding that can never be completely eradicated in observational studies justifies exercising of caution in drawing inferences of causality. These limitations underscore the need for additional validation in prospective cohort studies and in *vitro* or in *vivo* studies with regard to metabolism–inflammation–cardiovascular outcome relationships.

## Conclusions

The findings of this study suggest a strong relationship between metabolic parameters (TyG and AIP) and inflammation-related measures (DII and SIRI) with an increased prevalence of CVD, as well as higher risks of mortality. Notably, using the DII, these relationships between metabolic parameters and adverse cardiovascular outcomes were variably mediated. Furthermore, adults with both metabolic abnormalities and a high inflammatory burden in the upper tertile had the greatest prevalence of CVD and mortality risk. Clinically, these indicators—derived from routine laboratory tests and dietary information—are anticipated to supplement existing risk assessments, facilitating the identification of individuals at heightened risk owing to “metabolic abnormalities combined with a high inflammatory burden” during community health check-ups and outpatient visits. This approach enables earlier and more comprehensive management of risk factors including lipid levels and blood glucose, as well as weight management and healthy dietary interventions, thereby reducing the incidence of premature deaths attributed to CVD. This study provides population-level evidence that underscores the importance of prioritizing healthy dietary practices and comprehensive management interventions in phenotypically high-risk populations. Future research should focus on validating the reproducibility of risk stratification and thresholds in external populations, assessing whether the incorporation of these indicators into predictive models enhances risk assessment, and investigating through prospective and interventional studies whether a “combined metabolic management + anti-inflammatory diet” approach can yield clinical benefits.

## Supplementary Information

Supplementary Material 1

## Data Availability

The datasets used and evaluated in this study are available from the corresponding author upon reasonable request.

## Abbreviations

CVD: Cardiovascular disease
TyG: Triglyceride–glucose index
IR: Insulin resistance
AIP: Atherogenic index of plasma
Sd LDL: Small dense low-density lipoprotein
DII: Dietary inflammation index
SIRI: Systemic inflammatory response index
MEC: Mobile Examination Center
MCQ: Medical Conditions questionnaire
NDI: National Death Index
TG: Triglyceride
NHANES: National Health and Nutrition Examination Survey
BMI: Body mass index
FPG: Fasting plasma glucose
PIR: Poverty income ratio
HDL-C: High density lipoprotein cholesterol
MET: Metabolic equivalent
OR: Odds ratio
CI: Confidence interval
HR: Hazard ratio
ROC: Receiver operating characteristic
AUC: Area under the receiver operating characteristic curve
RCS: Restricted cubic splines
FDR: False discovery rate
