# Supplementary material for: Associations of metabolic indicators and inflammation-related indices with adverse cardiovascular events in US adults: NHANES 1999–2018
Source: Lipids Health Dis. 2026 Jan 21;25:57. doi: 10.1186/s12944-026-02866-w (PMC12905991; doi:10.1186/s12944-026-02866-w)
Supplement: Supplementary file 1 — Supplementary Material 1 [file 12944_2026_2866_MOESM1_ESM.docx]

**Supplemental Online Content**

**Fig. S1** Spearman's rank correlation coefficients between dietary quality indices, inﬂammation-related indices and metabolic indicators.

**Fig. S2** Associations of inﬂammation-related indices and metabolic indicators with CVD

**Fig. S3** Generalized additive models (GAM) illustrating the association of metabolic indicators and inﬂammation-related indices with CVD

**Fig. S4** The ROC curves display the sensitivity and specificity of each model in predicting mortality and identifying CVD prevalence

**Fig. S5** The ROC curves of TyG, DII, AIP and SIRI for predicting mortality and identifying CVD prevalence

**Fig. S6** Associations of metabolic indicators with CVD prevalence by inﬂammation-related indices.

**Fig. S7** Associations of metabolic indicators with mortality by inﬂammation-related indices.

**Fig. S8** Joint associations of metabolic indicators and inﬂammation-related indices with CVD prevalence.

**Table S1** Baseline characteristics those included and excluded participants.

**Table S2** Associations of Metabolic indicators with DII

**Table S3** Associations of Metabolic indicators with SIRI

**Table S4** Associations of Metabolic indicators with neutrophils, monocyte and lymphocyte number

**Table S5** Associations of inﬂammation-related indices and metabolic indicators with CVD in model 1 and 2

**Table S6** Associations of inﬂammation-related indices and metabolic indicators with mortality in model 1 and 2

**Table S7** Inflammation-related indices mediate the association of metabolic indicators with CVD and mortality

**Table S8** Components of SIRI mediate the association of metabolic indicators with CVD and mortality

**Table S9** Associations of inflammation-related indices and metabolic indicators with CVD prevalence, all-cause mortality, and cardiovascular mortality after multiple imputation

**Table S10** Inflammation-related indices mediate the association of metabolic indicators with CVD and mortality after multiple imputation

**Table S11** Joint associations of metabolic indicators and inﬂammation-related indices with CVD prevalence after multiple imputation

|  |
| --- |
| **Fig. S1** Spearman's rank correlation coefficients between inﬂammation-related indices and metabolic indicators. Note: Asterisks indicate the correlation is statistically significant. Panel A illustrates the Spearman rank-correlation coefficients between inflammatory markers and metabolic parameters in the entire cohort; Panel B presents the same correlations within the control group, and Panel C presents those observed in participants with cardiovascular disease (CVD). Abbreviations: CVD, Cardiovascular Disease; UA, Uric acid; LDL, Low-Density Lipoprotein; HDL, High-Density Lipoprotein; TC, Total Cholesterol; TG, Triglyceride; SIRI, System Inflammation Response Index; DII, Dietary Inflammatory Index; TyG, Triglyceride Glucose Index; AIP, Atherogenic index of plasma. |

| 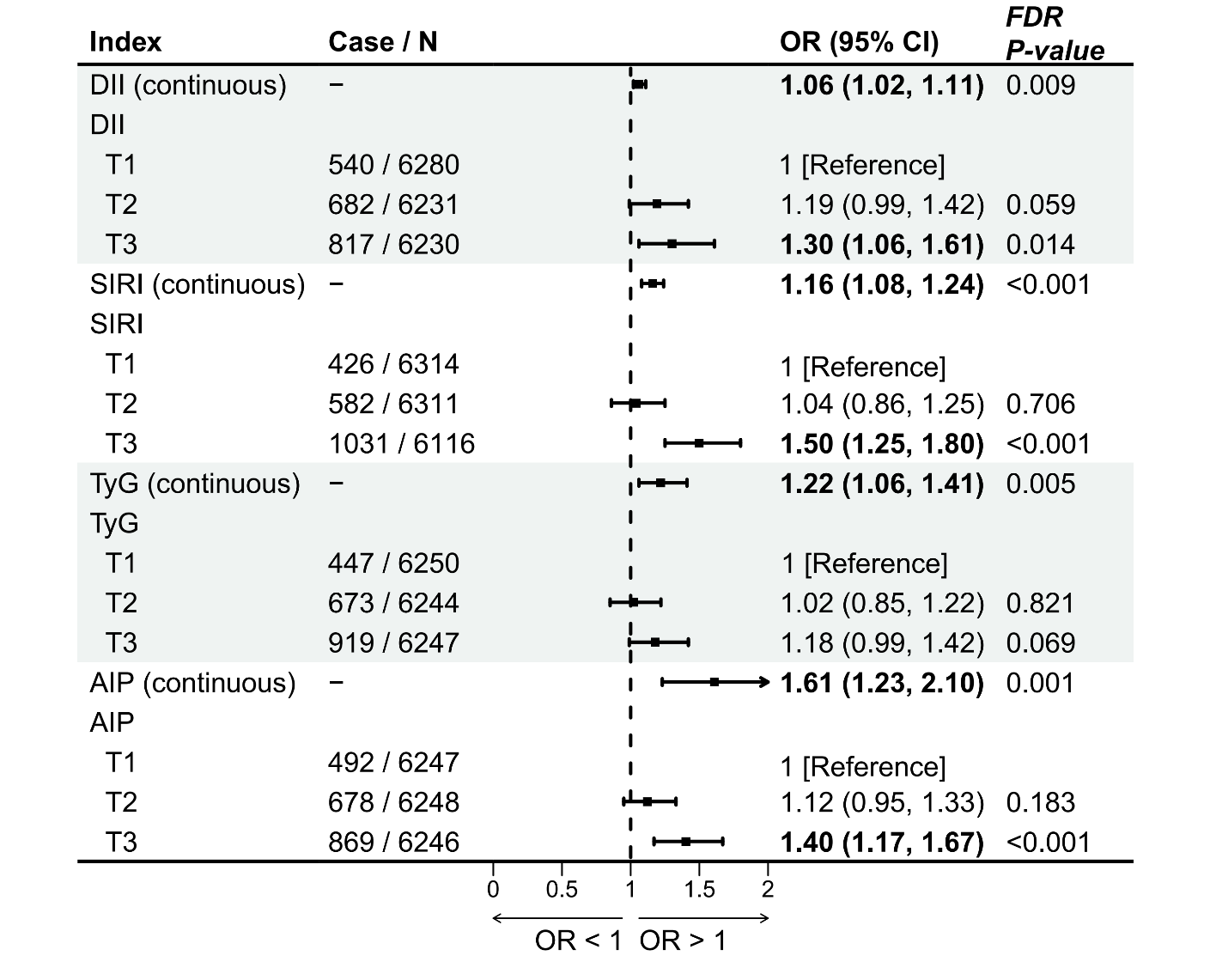 |
| --- |
| **Fig. S2** Associations of inﬂammation-related indices and metabolic indicators with CVD. The model was adjusted for gender, age, ethnic, economic level, BMI, hypertension, smoking status, energy intake and exercise status. The figure showed the *P*-values corrected by FDR. Abbreviations: DII, Dietary Inflammatory Index; SIRI, System Inflammation Response Index; TyG, Triglyceride Glucose Index; AIP, Atherogenic index of plasma; BMI, body mass index; OR, Odds ratio; CI, confidence interval; T, Tertile. ORs with statistical significance (*P* < 0.05) are presented in bold. |

|  |
| --- |
| **Fig. S3** Generalized additive models (GAM) illustrating the association of metabolic indicators and inﬂammation-related indices with CVD. The Model was adjusted by gender, age, ethnic, economic level, BMI, hypertension, smoking status, energy intake and exercise status. The vertical line of the dotted line is the inflection point where the risk changes from "nonsignificant" to "significant". Abbreviations: CVD, Cardiovascular Disease; SIRI, System Inflammation Response Index; DII, Dietary Inflammatory Index; TyG, Triglyceride Glucose Index; AIP, Atherogenic index of plasma; BMI, body mass index; OR, Odds ratio; CI, confidence interval. |

|  |
| --- |
| **Fig. S4** The ROC curves display the sensitivity and specificity of each model in predicting mortality and identifying CVD prevalence. The AUC represents the discrimination ability of each model. A, E, I: TyG index; B, F, J: AIP index; C, G, H: DII index; D, H, L: SIRI index. Model 1: Adjusted for gender, age, ethnic, and economic level; Model 2: additionally adjusted for BMI and hypertension in Model 1; Model 3: average energy intake, smoking status and exercise status were further adjusted in Model 2. Abbreviations: ROC, receiver operating characteristic; AUC, area under the curve; CVD, cardiovascular disease; SIRI, System Inflammation Response Index; DII, Dietary Inflammatory Index; TyG, Triglyceride Glucose Index; AIP, Atherogenic index of plasma. |

|  |
| --- |
| **Fig. S5** The ROC curves of TyG, DII, AIP and SIRI for predicting mortality and identifying CVD prevalence. The AUC represents the discrimination ability of each index. A: CVD prevalence; B: All-cause mortality; C: CVD mortality. Abbreviations: ROC, receiver operating characteristic; AUC, area under the curve; CVD, cardiovascular disease; SIRI, System Inflammation Response Index; DII, Dietary Inflammatory Index; TyG, Triglyceride Glucose Index; AIP, Atherogenic index of plasma. |

|  |
| --- |
| **Fig. S6** Associations of metabolic indicators with CVD prevalence by inﬂammation-related indices. Odd ratios were adjusted for gender, age, ethnic, economic level, BMI, hypertension, smoking status, energy intake and exercise status. Multiplicative interaction was evaluated using odd ratios for the product term between the metabolic indicators (T1, T2, T3) and inﬂammation-related indices (T1, T2, T3), and the multiplicative interaction was statistically significant when its CI did not include 1. Additive interaction was evaluated using relative excess risk due to interaction (RERI) between the metabolic indicators (T1, T2, T3) and inﬂammation-related indices (T1, T2, T3), and the additive interaction was statistically significant when its CI did not include 0. Due to multiple comparisons being performed, both the *P*-values and the FDR-adjusted *P*-values were provided. Abbreviations: CVD, Cardiovascular Disease; SIRI, System Inflammation Response Index; DII, Dietary Inflammatory Index; TyG, Triglyceride Glucose Index; AIP, Atherogenic index of plasma; BMI, body mass index; OR, Odds ratio; CI, confidence interval; T, Tertile. |

|  |
| --- |
| **Fig. S7** Associations of metabolic indicators with mortality by inﬂammation-related indices. Hazard ratios were adjusted for gender, age, ethnic, economic level, BMI, hypertension, smoking status, energy intake and exercise status. Multiplicative interaction was evaluated using hazard ratios for the product term between the metabolic indicators (T1, T2, T3) and inﬂammation-related indices (T1, T2, T3), and the multiplicative interaction was statistically significant when its CI did not include 1. Additive interaction was evaluated using relative excess risk due to interaction (RERI) between the metabolic indicators (T1, T2, T3) and inﬂammation-related indices (T1, T2, T3), and the additive interaction was statistically significant when its CI did not include 0. Due to multiple comparisons being performed, both the *P*-values and the FDR-adjusted *P*-values were provided. Abbreviations: CVD, Cardiovascular Disease; SIRI, System Inflammation Response Index; DII, Dietary Inflammatory Index; TyG, Triglyceride Glucose Index; AIP, Atherogenic index of plasma; BMI, body mass index; HR, Hazard ratio; CI, confidence interval; T, Tertile. |

|  |
| --- |
| **Fig. S8** Joint associations of metabolic indicators and inﬂammation-related indices with CVD prevalence. ORs were adjusted for gender, age, ethnic, economic level, BMI, hypertension, smoking status, energy intake and exercise status. Due to multiple comparisons being performed, both the *P*-values and the FDR-adjusted *P*-values were provided. Abbreviations: CVD, Cardiovascular Disease; SIRI, System Inflammation Response Index; DII, Dietary Inflammatory Index; TyG, Triglyceride Glucose Index; AIP, Atherogenic index of plasma; BMI, body mass index; OR, Odds ratio; CI, confidence interval; T, Tertile. |

**Table S1** Baseline characteristics those included and excluded participants.

| **Characteristics** | **Excluded**  **(N =** **4630)** | **Included**  **(N = 18741)** | ***P* value** |
| --- | --- | --- | --- |
| **General characteristics** |  |  |  |
| **Gender (%)** |  |  | 0.046 |
| Male | 2324 (50.6) | 9208 (48.5) |  |
| Female | 2306 (49.4) | 9533 (51.5) |  |
| **Age, y, mean (SD)** | 41.89 (20.17) | 46.90 (16.68) | <0.001 |
| **Race (%)** |  |  | <0.001 |
| Mexican American | 1019 (9.3) | 3299 (8.1) |  |
| Non-Hispanic Black | 1090 (13.6) | 3648 (10.8) |  |
| Non-Hispanic White | 1663 (61.0) | 8522 (69.2) |  |
| Other | 858 (16.0) | 3272 (11.9) |  |
| **Marital (%)** |  |  | <0.001 |
| Yes | 1711 (43.0) | 11531 (65.0) |  |
| No | 2919 (57.0) | 7210 (35.0) |  |
| **Income-to-poverty (%)** |  |  | <0.001 |
| Not poor | 3428 (81.2) | 15416 (87.6) |  |
| Poor | 1202 (18.8) | 3325 (12.4) |  |
| **Education status (%)** |  |  | 0.373 |
| Below high school | 1027 (17.5) | 4813 (16.8) |  |
| High School or above | 3603 (82.5) | 13928 (83.2) |  |
| **BMI, kg/m^2^, mean (SD)** | 28.04 (7.48) | 28.74 (6.67) | 0.001 |
| **Waist circumference, cm, mean (SD)** | 94.52 (16.84) | 98.41 (16.38) | <0.001 |
| **Abdominal obesity (%)** |  |  | <0.001 |
| Yes | 2222 (50.5) | 14162 (74.0) |  |
| No | 1594 (30.3) | 4579 (26.0) |  |
| Missing | 814 (19.2) | 0 (0.0) |  |
| **Average energy intake, kcal, mean (SD)** | 0.00 (0.15) | 0.00 (0.15) | 0.558 |
| **Smoke (%)** |  |  | <0.001 |
| Yes | 1406 (36.9) | 8579 (46.4) |  |
| No | 2538 (56.1) | 10162 (53.6) |  |
| Missing | 686 (7.0) | 0 (0.0) |  |
| **Alcohol (%)** |  |  | <0.001 |
| Yes | 1837 (50.4) | 13652 (77.6) |  |
| No | 518 (10.1) | 2228 (9.6) |  |
| Missing | 2275 (39.5) | 2861 (12.8) |  |
| **Physical activity (%)** |  |  | <0.001 |
| Light physical activity | 2716 (55.9) | 9437 (47.1) |  |
| High level of physical activity | 1914 (44.1) | 9304 (52.9) |  |
| **Diabetes (%)** |  |  | <0.001 |
| Yes | 681 (12.7) | 3127 (12.5) |  |
| No | 3354 (69.4) | 15614 (87.5) |  |
| Missing | 595 (17.9) | 0 (0.0) |  |
| **Hypertension (%)** |  |  | <0.001 |
| Yes | 1524 (32.7) | 7865 (36.7) |  |
| No | 3102 (67.1) | 10876 (63.3) |  |
| Missing | 4 (0.1) | 0 (0.0) |  |
| **Laboratory characteristics** |  |  |  |
| **Neutrophils number, mean (SD), 10^9^/L** | 4.04 (1.62) | 3.99 (1.59) | 0.174 |
| **Lymphocyte number, mean (SD), 10^9^/L** | 2.04 (0.88) | 2.00 (1.02) | 0.061 |
| **Monocyte number, mean (SD), 10^9^/L** | 0.54 (0.19) | 0.54 (0.19) | 0.185 |
| **HbA1c, %, mean (SD)** | 5.54 (1.10) | 5.56 (0.87) | 0.536 |
| **FPG, mmol/L, mean (SD)** | 5.88 (2.18) | 5.80 (1.54) | 0.158 |
| **LDL-cholesterol, mmol/L, mean (SD)** | 2.84 (0.93) | 2.99 (0.91) | <0.001 |
| **HDL-cholesterol, mmol/L, mean (SD)** | 1.32 (0.42) | 1.39 (0.41) | <0.001 |
| **TC, mmol/L, mean (SD)** | 5.03 (1.31) | 5.01 (1.03) | 0.651 |
| **TG, mmol/L, mean (SD)** | 2.07 (2.80) | 1.36 (0.76) | <0.001 |
| **Uric acid, umol/L, mean (SD)** | 330.09 (88.99) | 324.70 (82.48) | 0.021 |
| **Inﬂammation-related indices** |  |  |  |
| **SIRI, mean (SD)** | 0.99 [0.67, 1.46] | 1.00 [0.69, 1.44] | 0.625 |
| **DII, mean (SD)** | 1.88 [0.33, 2.95] | 1.68 [0.15, 2.91] | 0.131 |
| **Metabolic indicators** |  |  |  |
| **TyG, mean (SD)** | 8.74 (0.92) | 8.58 (0.61) | <0.001 |
| **AIP, mean (SD)** | 0.04 (0.44) | -0.06 (0.31) | <0.001 |

**Note:** Continuous variable with a normal or skewed distribution was presented as mean (SD) or median (25th-75th percentile), and categorical variables were presented as numbers (percentages). Variables between groups were compared by variance test, Kruskal-Wallis test, or Chi-square test.

**Abbreviations:** BMI, body mass index; HbA1c, Hemoglobin A1c; FPG, Fasting Plasma Glucose; LDL, Low-Density Lipoprotein; HDL, High-Density Lipoprotein; TC, Total Cholesterol; TG, Triglyceride; SIRI, System Inflammation Response Index; DII, Dietary Inflammatory Index; TyG, Triglyceride Glucose Index; AIP, Atherogenic index of plasma; SD, standard deviation.

**Table S2** Associations of Metabolic indicators with DII

| **Metabolic indicators** | **β** | **95% CI** | ***P* value** |
| --- | --- | --- | --- |
| **Model 1** |  |  |  |
| TyG | **0.25** | **(0.19, 0.32)** | <0.001 |
| AIP | **0.60** | **(0.48, 0.72)** | <0.001 |
| **Model 2** |  |  |  |
| TyG | **0.19** | **(0.13, 0.26)** | <0.001 |
| AIP | **0.49** | **(0.37, 0.62)** | <0.001 |
| **Model 3** |  |  |  |
| TyG | **0.12** | **(0.07, 0.18)** | <0.001 |
| AIP | **0.33** | **(0.22, 0.43)** | <0.001 |

**Note:** Model 1: Adjusted for gender, age, ethnic, and economic level; Model 2: additionally adjusted for BMI and hypertension in Model 1; Model 3: average energy intake, smoking status and exercise status were further adjusted in Model 2. Abbreviations: DII, Dietary Inflammatory Index; β, Beta value; CI, confidence interval; TyG, Triglyceride Glucose Index; AIP, Atherogenic index of plasma; BMI, body mass index. β with statistical significance (*P* < 0.05) was presented in bold.

**Table S3** Associations of Metabolic indicators with SIRI

| **Metabolic indicators** | **β** | **95% CI** | ***P* value** |
| --- | --- | --- | --- |
| **Model 1** |  |  |  |
| TyG | **0.03** | **(0.00, 0.06)** | 0.025 |
| AIP | **0.08** | **(0.02, 0.13)** | 0.007 |
| **Model 2** |  |  |  |
| TyG | -0.01 | (-0.04, 0.02) | 0.600 |
| AIP | 0.01 | (-0.06, 0.07) | 0.900 |
| **Model 3** |  |  |  |
| TyG | -0.03 | (-0.06, 0.00) | 0.080 |
| AIP | -0.04 | (-0.11, 0.03) | 0.200 |

Note: Model 1: Adjusted for gender, age, ethnic, and economic level; Model 2: additionally adjusted for BMI and hypertension in Model 1; Model 3: average energy intake, smoking status and exercise status were further adjusted in Model 2. Abbreviations: SIRI, System Inflammation Response Index; β, Beta value; CI, confidence interval; TyG, Triglyceride Glucose Index; AIP, Atherogenic index of plasma; BMI, body mass index. β with statistical significance (*P* < 0.05) was presented in bold.

**Table S4** Associations of metabolic indicators with neutrophils, monocyte and lymphocyte number

| **Metabolic indicators** | **β** | **95% CI** | ***P* value** |
| --- | --- | --- | --- |
| **Neutrophils** **number** |  |  |  |
| TyG | **0.32** | **(0.27, 0.38)** | <0.001 |
| AIP | 0.65 | (0.54, 0.76) | 0.65 |
| **Monocyte** **number** |  |  |  |
| TyG | **0.01** | **(0.01, 0.02)** | <0.001 |
| AIP | **0.04** | **(0.03, 0.05)** | <0.001 |
| **Lymphocyte** **number** |  |  |  |
| TyG | **0.22** | **(0.19, 0.25)** | <0.001 |
| AIP | **0.46** | **(0.40, 0.52)** | <0.001 |

Note: Adjusted for gender, age, ethnic, economic level, BMI, hypertension, smoking status, energy intake and exercise status. Abbreviations: TyG, Triglyceride Glucose Index; AIP, Atherogenic index of plasma; BMI, body mass index; CI, confidence interval. β with statistical significance (*P* < 0.05) was presented in bold.

**Table S5** Associations of inﬂammation-related indices and metabolic indicators with CVD in model 1 and 2

| **Index** | **Case / N** | **OR** | **95% CI** | ***P* value** |
| --- | --- | --- | --- | --- |
| **Model 1** |  |  |  |  |
| DII (continuous) | **-** | **1.13** | **(1.08, 1.17)** | <0.001 |
| DII |  |  |  | <0.001 |
| T1 | 540 / 6280 | 1 [Reference] | 1 [Reference] |  |
| T2 | 682 / 6231 | **1.31** | **(1.11, 1.54)** |  |
| T3 | 817 / 6230 | **1.64** | **(1.37, 1.97)** |  |
| SIRI (continuous) | - | **1.23** | **(1.15, 1.32)** | <0.001 |
| SIRI |  |  |  | <0.001 |
| T1 | 426 / 6314 | 1 [Reference] | 1 [Reference] |  |
| T2 | 582 / 6311 | 1.13 | (0.93, 1.36) |  |
| T3 | 1031 / 6116 | **1.76** | **(1.47, 2.11)** |  |
| TyG (continuous) | **-** | **1.51** | **(1.33, 1.73)** | <0.001 |
| TyG |  |  |  | <0.001 |
| T1 | 447 / 6250 | 1 [Reference] | 1 [Reference] |  |
| T2 | 673 / 6244 | 1.16 | (0.98, 1.38) |  |
| T3 | 919 / 6247 | **1.60** | **(1.35, 1.90)** |  |
| AIP (continuous) | - | **2.37** | **(1.83, 3.07)** | <0.001 |
| AIP |  |  |  | <0.001 |
| T1 | 492 / 6247 | 1 [Reference] | 1 [Reference] |  |
| T2 | 678 / 6248 | **1.30** | **(1.10, 1.52)** |  |
| T3 | 869 / 6246 | **1.85** | **(1.56, 2.20)** |  |
| **Model 2** |  |  |  |  |
| DII (continuous) | **-** | **1.11** | **(1.07, 1.16)** | <0.001 |
| DII |  |  |  | <0.001 |
| T1 | 540 / 6280 | 1 [Reference] | 1 [Reference] |  |
| T2 | 682 / 6231 | **1.28** | **(1.08, 1.53)** |  |
| T3 | 817 / 6230 | **1.56** | **(1.30, 1.87)** |  |
| SIRI (continuous) | - | **1.19** | **(1.12, 1.28)** | <0.001 |
| SIRI |  |  |  | <0.001 |
| T1 | 426 / 6314 | 1 [Reference] | 1 [Reference] |  |
| T2 | 582 / 6311 | 1.06 | (0.88, 1.28) |  |
| T3 | 1031 / 6116 | **1.59** | **(1.32, 1.91)** |  |
| TyG (continuous) | **-** | **1.28** | **(1.11, 1.47)** | <0.001 |
| TyG |  |  |  | <0.001 |
| T1 | 447 / 6250 | 1 [Reference] | 1 [Reference] |  |
| T2 | 673 / 6244 | 1.04 | (0.87, 1.25) |  |
| T3 | 919 / 6247 | **1.26** | **(1.05, 1.52)** |  |
| AIP (continuous) | - | **1.77** | **(1.35, 2.32)** | <0.001 |
| AIP |  |  |  | <0.001 |
| T1 | 492 / 6247 | 1 [Reference] | 1 [Reference] |  |
| T2 | 678 / 6248 | 1.16 | (0.98, 1.37) |  |
| T3 | 869 / 6246 | **1.50** | **(1.25, 1.79)** |  |

Note: Model 1: Adjusted for gender, age, ethnic, and economic level; Model 2: additionally adjusted for BMI and hypertension in Model 1. Abbreviations: DII, Dietary Inflammatory Index; SIRI, System Inflammation Response Index; TyG, Triglyceride Glucose Index; AIP, Atherogenic index of plasma; BMI, body mass index; OR, Odds ratio; CI, confidence interval; T, Tertile. ORs with statistical significance (*P* < 0.05) are presented in bold.

**Table S6** Associations of inﬂammation-related indices and metabolic indicators with mortality in model 1 and 2

| **Index** | **Case / N** | **HR** | **95% CI** | ***P* value** |
| --- | --- | --- | --- | --- |
| **All-cause mortality** | | | | |
| **Model 1** |  |  |  |  |
| DII (continuous) | **-** | **1.14** | **(1.11, 1.17)** | <0.001 |
| DII |  |  |  | <0.001 |
| T1 | 722 / 6280 | 1 [Reference] | 1 [Reference] |  |
| T2 | 934 / 6231 | **1.36** | **(1.22, 1.51)** |  |
| T3 | 958 / 6230 | **1.62** | **(1.45, 1.81)** |  |
| SIRI (continuous) | - | **1.21** | **(1.15, 1.28)** | <0.001 |
| SIRI |  |  |  | <0.001 |
| T1 | 513 / 6314 | 1 [Reference] | 1 [Reference] |  |
| T2 | 751 / 6311 | 1.07 | (0.93, 1.25) |  |
| T3 | 1350 / 6116 | **1.54** | **(1.34, 1.77)** |  |
| TyG (continuous) | **-** | **1.27** | **(1.15, 1.39)** | <0.001 |
| TyG |  |  |  | <0.001 |
| T1 | 521 / 6250 | 1 [Reference] | 1 [Reference] |  |
| T2 | 915 / 6244 | 1.04 | (0.92, 1.17) |  |
| T3 | 1178 / 6247 | **1.26** | **(1.10, 1.44)** |  |
| AIP (continuous) | - | **1.40** | **(1.15, 1.70)** | <0.001 |
| AIP |  |  |  | <0.001 |
| T1 | 641 / 6247 | 1 [Reference] | 1 [Reference] |  |
| T2 | 915 / 6248 | 1.07 | (0.94, 1.21) |  |
| T3 | 1058 / 6246 | **1.24** | **(1.10, 1.40)** |  |
| **Model 2** |  |  |  |  |
| DII (continuous) | **-** | **1.13** | **(1.11, 1.16)** | <0.001 |
| DII |  |  |  | <0.001 |
| T1 | 722 / 6280 | 1 [Reference] | 1 [Reference] |  |
| T2 | 934 / 6231 | **1.35** | **(1.22, 1.50)** |  |
| T3 | 958 / 6230 | **1.59** | **(1.42, 1.77)** |  |
| SIRI (continuous) | - | **1.21** | **(1.14, 1.28)** | <0.001 |
| SIRI |  |  |  | <0.001 |
| T1 | 513 / 6314 | 1 [Reference] | 1 [Reference] |  |
| T2 | 751 / 6311 | 1.07 | (0.92, 1.23) |  |
| T3 | 1350 / 6116 | 1.52 | **(1.32, 1.75)** |  |
| TyG (continuous) | **-** | 1.25 | **(1.14, 1.38)** | <0.001 |
| TyG |  |  |  | <0.001 |
| T1 | 521 / 6250 | 1 [Reference] | 1 [Reference] |  |
| T2 | 915 / 6244 | 1.04 | (0.92, 1.16) |  |
| T3 | 1178 / 6247 | 1.24 | **(1.09, 1.41)** |  |
| AIP (continuous) | - | 1.37 | **(1.13, 1.67)** | 0.002 |
| AIP |  |  |  | 0.002 |
| T1 | 641 / 6247 | 1 [Reference] | 1 [Reference] |  |
| T2 | 915 / 6248 | 1.07 | (0.94, 1.22) |  |
| T3 | 1058 / 6246 | **1.22** | **(1.08, 1.38)** |  |
| **CVD mortality** | | | | |
| **Model 1** |  |  |  |  |
| DII (continuous) | **-** | **1.15** | **(1.10, 1.21)** | <0.001 |
| DII |  |  |  | <0.001 |
| T1 | 187 / 6280 | 1 [Reference] | 1 [Reference] |  |
| T2 | 235 / 6231 | 1.21 | (0.98, 1.49) |  |
| T3 | 255 / 6230 | **1.78** | **(1.46, 2.17)** |  |
| SIRI (continuous) | - | **1.23** | **(1.14, 1.32)** | <0.001 |
| SIRI |  |  |  | <0.001 |
| T1 | 111 / 6314 | 1 [Reference] | 1 [Reference] |  |
| T2 | 184 / 6311 | 1.19 | (0.86, 1.63) |  |
| T3 | 382 / 6116 | **2.00** | **(1.41, 2.85)** |  |
| TyG (continuous) | **-** | **1.44** | **(1.15, 1.81)** | 0.001 |
| TyG |  |  |  | 0.015 |
| T1 | 125 / 6250 | 1 [Reference] | 1 [Reference] |  |
| T2 | 237 / 6244 | 1.09 | (0.83, 1.43) |  |
| T3 | 315 / 6247 | **1.39** | **(1.06, 1.82)** |  |
| AIP (continuous) | - | **1.62** | **(1.08, 2.43)** | 0.021 |
| AIP |  |  |  | 0.066 |
| T1 | 154 / 6247 | 1 [Reference] | 1 [Reference] |  |
| T2 | 234 / 6248 | 1.17 | (0.93, 1.48) |  |
| T3 | 289 / 6246 | **1.36** | **(1.05, 1.76)** |  |
| **Model 2** |  |  |  |  |
| DII (continuous) | **-** | **1.14** | **(1.08, 1.20)** | <0.001 |
| DII |  |  |  | <0.001 |
| T1 | 187 / 6280 | 1 [Reference] | 1 [Reference] |  |
| T2 | 235 / 6231 | 1.18 | (0.97, 1.45) |  |
| T3 | 255 / 6230 | **1.70** | **(1.39, 2.09)** |  |
| SIRI (continuous) | - | **1.22** | **(1.13, 1.32)** | <0.001 |
| SIRI |  |  |  | <0.001 |
| T1 | 111 / 6314 | 1 [Reference] | 1 [Reference] |  |
| T2 | 184 / 6311 | 1.16 | (0.84, 1.59) |  |
| T3 | 382 / 6116 | **1.93** | **(1.36, 2.74)** |  |
| TyG (continuous) | **-** | **1.32** | **(1.04, 1.68)** | 0.024 |
| TyG |  |  |  | 0.2 |
| T1 | 125 / 6250 | 1 [Reference] | 1 [Reference] |  |
| T2 | 237 / 6244 | 1.04 | (0.78, 1.37) |  |
| T3 | 315 / 6247 | 1.23 | (0.93, 1.63) |  |
| AIP (continuous) | - | 1.37 | (0.89, 2.11) | 0.150 |
| AIP |  |  |  | 0.4 |
| T1 | 154 / 6247 | 1 [Reference] | 1 [Reference] |  |
| T2 | 234 / 6248 | 1.12 | (0.89, 1.42) |  |
| T3 | 289 / 6246 | 1.21 | (0.93, 1.59) |  |

Note: Model 1: Adjusted for gender, age, ethnic, and economic level; Model 2: additionally adjusted for BMI and hypertension in Model 1. Abbreviations: DII, Dietary Inflammatory Index; SIRI, System Inflammation Response Index; TyG, Triglyceride Glucose Index; AIP, Atherogenic index of plasma; BMI, body mass index; HR, Hazard ratio; CI, confidence interval; T, Tertile. HRs with statistical significance (*P* < 0.05) are presented in bold.

**Table S7** Inflammation-related indices mediate the association of metabolic indicators with CVD and mortality

| M/X | Outcome | TyG | AIP |
| --- | --- | --- | --- |
| **CVD prevalence** | | | |
| DII | ACME | 0.000139 (0.000135, 0.000142)*** | 0.00146 (0.001440, 0.001485)*** |
|  | ADE | 0.00374 (0.003719, 0.003758)*** | 0.0351 (0.034746, 0.035501) *** |
|  | Prop. Mediated(%) | 3.58% (3.50%, 3.66%)*** | 3.99% (3.93%, 4.07%) *** |
| SIRI | ACME | -0.000081 (-0.000083, -0.000079) *** | -0.00046(-0.00047, -0.00045) *** |
|  | ADE | 0.00393 (0.003908, 0.003956) *** | 0.0374 (0.037014 , 0.037739) *** |
|  | Prop. Mediated(%) | -2.09% (-2.14%, -2.05%) *** | -1.25% (-1.28%, -1.21%) *** |
| **All-cause morality** | | | |
| SIRI | ACME | 0.9952 (0.9881, 0.9999) * | 0.9932 (0.9809, 1.0038) |
|  | ADE | 1.2110 (1.0969, 1.3497) *** | 1.2988 (1.0680, 1.5816) ** |
|  | Prop. Mediated(%) | -2.84% (-10.36, -0.03) * | -3.05 (-17.00, 2.11) |
| **CVD morality** | | | |
| SIRI | ACME | 0.9948 (0.9848, 0.9999) * | 0.9926 (0.9771, 1.0043) |
|  | ADE | 1.2977 (1.0311, 1.6289) * | 1.3380 (0.9096, 1.9042) |
|  | Prop. Mediated(%) | -2.34% (-12.98%, 0.51%) | -3.02% (-31.67%, 23.23%) |

Note: Adjusted for gender, age, ethnic, economic level, BMI, hypertension, smoking status, energy intake and exercise status. Abbreviations: CVD, Cardiovascular Disease; SIRI, System Inflammation Response Index; DII, Dietary Inflammatory Index; TyG, Triglyceride Glucose Index; AIP, Atherogenic index of plasma; BMI, body mass index; CI, confidence interval. ACME, average causal mediation effects (indirect effect); ADE, average direct effects. * *P* < 0.05, ** *P* < 0.01, and *** *P* < 0.001.

**Table S8** Components of SIRI mediate the association of metabolic indicators with CVD and mortality

| M/X | Outcome | TyG | AIP |
| --- | --- | --- | --- |
| **CVD prevalence** | | | |
| Neutrophils number | ACME | 0.000458 (0.000448,  0.000466)*** | 0.00364(0.003606,0.003669)*** |
|  | ADE | 0.00343 (0.003409,0.003441)*** | 0.0328(0.032475, 0.033201) *** |
|  | Prop. Mediated(%) | 11.80% (11.60%, 11.96%)*** | 9.98% (9.86%, 10.09%) *** |
| Monocyte number | ACME | 0.000121(0.000118,0.000125)*** | 0.00134(0.001313,0.001373)*** |
|  | ADE | 0.00376(0.003738, 0.003779)*** | 0.0346 (0.034257, 0.035003)*** |
|  | Prop. Mediated(%) | 3.13% (3.05%, 3.21%) *** | 3.73% (3.63%, 3.83%)*** |
| Lymphocyte number | ACME | 0.0000000885 (-0.000001,  0.000001) | -0.0000871 (-0.000094,  -0.000080) *** |
|  | ADE | 0.00389(0.003872, 0.003914)*** | 0.0361(0.035754, 0.036505)*** |
|  | Prop. Mediated(%) | 0.0023% (-0.020%, 0.024%) | -0.24% (-0.26%, -0.22%) *** |
| **All-cause morality** | | | |
| Neutrophils number | ACME | 1.0294 (1.0230, 1.0521)*** | 1.0605 (1.0472, 1.1097)*** |
|  | ADE | 1.1668 (1.0515, 1.2901)*** | 1.1846 (0.9695, 1.4260) |
|  | Prop. Mediated(%) | 17.05% (10.97%, 42.55%)*** | 27.96% (14.78%, 114.66%)* |
| Monocyte number | ACME | 1.0044 (1.0024, 1.0128)*** | 1.0127 (1.0076, 1.0358)*** |
|  | ADE | 1.1948 (1.0778, 1.3252)*** | 1.2340 (1.0061, 1.4838)* |
|  | Prop. Mediated(%) | 2.63% (1.22%, 8.83%)*** | 6.29% (2.62%, 41.26%)* |
| Lymphocyte number | ACME | 1.0047 (0.9983, 1.0091) | 1.0100 (0.9975, 1.0191) |
|  | ADE | 1.1966 (1.0777, 1.3261)*** | 1.2477 (1.0212, 1.5014)* |
|  | Prop. Mediated(%) | 2.77% (-1.27%, 7.33%) | 4.80% (-2.10%, 24.09%) |
| **CVD morality** | | | |
| Neutrophils number | ACME | 1.0294 (1.0219, 1.0674)*** | 1.0607 (1.0460, 1.1435)*** |
|  | ADE | 1.2498 (0.9848, 1.5534) | 1.2031 (0.8173, 1.6836) |
|  | Prop. Mediated(%) | 12.83 (6.05%, 69.74%)* | 26.43% (-199.97%, 263.24%) |
| Monocyte number | ACME | 1.0037 (1.0014, 1.0135)*** | 1.0107 (1.0042, 1.0391)*** |
|  | ADE | 1.2803 (1.0230, 1.5874)* | 1.2600 (0.8535, 1.7699) |
|  | Prop. Mediated(%) | 1.66% (0.37%, 11.13%)* | 4.94% (-42.12%, 56.73%) |
| Lymphocyte number | ACME | 0.9717 (0.9320, 0.9961)** | 0.9471 (0.8688, 0.9935)* |
|  | ADE | 1.3158 (1.0492, 1.6407)* | 1.3492 (0.9304, 1.9249) |
|  | Prop. Mediated(%) | -13.35% (-70.30%, -0.55%)* | -25.71% (-328.23%, 171.08%) |

Note: Adjusted for gender, age, ethnic, economic level, BMI, hypertension, smoking status, energy intake and exercise status. Abbreviations: CVD, Cardiovascular Disease; SIRI, System Inflammation Response Index; TyG, Triglyceride Glucose Index; AIP, Atherogenic index of plasma; BMI, body mass index; CI, confidence interval; ACME, average causal mediation effects (indirect effect); ADE, average direct effects. * *P* < 0.05, ** *P* < 0.01, and *** *P* < 0.001.

**Table S9 Associations of inflammation-related indices and metabolic indicators with CVD prevalence, all-cause mortality, and cardiovascular mortality** **after multiple imputation**

| **Index** | **Case / N** | **OR/HR** | **95% CI** | ***FDR***  ***P-*value** |
| --- | --- | --- | --- | --- |
| **CVD prevalence** | | | | |
| DII (continuous) | **-** | **1.052** | **(1.007, 1.100)** | 0.024 |
| DII |  |  |  |  |
| T1 | 649 / 7202 | 1 [Reference] | 1 [Reference] |  |
| T2 | 814 / 7201 | 1.130 | (0.957, 1.334) | 0.148 |
| T3 | 988 / 7201 | **1.270** | **(1.046, 1.542)** | 0.016 |
| SIRI (continuous) | - | **1.153** | **(1.085, 1.226)** | <0.001 |
| SIRI |  |  |  |  |
| T1 | 518 / 7218 | 1 [Reference] | 1 [Reference] |  |
| T2 | 691 / 7188 | 1.053 | (0.885, 1.253) | 0.559 |
| T3 | 1242 / 7198 | **1.472** | **(1.234, 1.757)** | <0.001 |
| TyG (continuous) | **-** | **1.273** | **(1.145, 1.415)** | <0.001 |
| TyG |  |  |  |  |
| T1 | 532 / 7206 | 1 [Reference] | 1 [Reference] |  |
| T2 | 792 / 7199 | 1.028 | (0.865, 1.221) | 0.755 |
| T3 | 1127 / 7199 | **1.316** | **(1.110, 1.560)** | 0.002 |
| AIP (continuous) | - | **1.704** | **(1.376, 2.111)** | <0.001 |
| AIP |  |  |  |  |
| T1 | 587 / 7202 | 1 [Reference] | 1 [Reference] |  |
| T2 | 824 / 7203 | 1.145 | (0.984, 1.331) | 0.079 |
| T3 | 1040 / 7199 | **1.533** | **(1.301, 1.805)** | <0.001 |
| **All-cause mortality** | | | | |
| DII (continuous) | **-** | **1.114** | **(1.085, 1.144)** | <0.001 |
| DII |  |  |  |  |
| T1 | 915 / 7202 | 1 [Reference] | 1 [Reference] |  |
| T2 | 1158 / 7201 | **1.279** | **(1.160, 1.412)** | <0.001 |
| T3 | 1200 / 7201 | **1.449** | **(1.298, 1.617)** | <0.001 |
| SIRI (continuous) | - | 1.188 | (1.123, 1.257) | <0.001 |
| SIRI |  |  |  |  |
| T1 | 631 / 7218 | 1 [Reference] | 1 [Reference] |  |
| T2 | 924 / 7188 | 1.129 | (0.989, 1.289) | 0.073 |
| T3 | 1718 / 7198 | **1.570** | **(1.384, 1.781)** | <0.001 |
| TyG (continuous) | **-** | **1.172** | **(1.092, 1.257)** | <0.001 |
| TyG |  |  |  |  |
| T1 | 674 / 7206 | 1 [Reference] | 1 [Reference] |  |
| T2 | 1139 / 7199 | 0.951 | (0.856, 1.057) | 0.349 |
| T3 | 1460 / 7199 | **1.133** | **(1.016, 1.264)** | 0.024 |
| AIP (continuous) | - | **1.215** | **(1.042, 1.416)** | 0.013 |
| AIP |  |  |  |  |
| T1 | 823 / 7202 | 1 [Reference] | 1 [Reference] |  |
| T2 | 1145 / 7203 | 0.976 | (0.873, 1.092) | 0.676 |
| T3 | 1305 / 7199 | 1.094 | (0.979, 1.223) | 0.114 |
| **CVD mortality** | | | | |
| DII (continuous) | **-** | **1.127** | **(1.068, 1.190)** | <0.001 |
| DII |  |  |  |  |
| T1 | 235 / 7202 | 1 [Reference] | 1 [Reference] |  |
| T2 | 298 / 7201 | **1.217** | **(1.010, 1.466)** | 0.039 |
| T3 | 321 / 7201 | **1.619** | **(1.310, 2.002)** | <0.001 |
| SIRI (continuous) | - | **1.201** | **(1.116, 1.292)** | <0.001 |
| SIRI |  |  |  |  |
| T1 | 142 / 7218 | 1 [Reference] | 1 [Reference] |  |
| T2 | 228 / 7188 | 1.209 | (0.933, 1.566) | 0.152 |
| T3 | 484 / 7198 | **1.856** | **(1.394, 2.472)** | <0.001 |
| TyG (continuous) | **-** | **1.265** | **(1.081, 1.480)** | 0.003 |
| TyG |  |  |  |  |
| T1 | 160 / 7206 | 1 [Reference] | 1 [Reference] |  |
| T2 | 302 / 7199 | 0.997 | (0.783, 1.268) | 0.977 |
| T3 | 392 / 7199 | 1.185 | (0.937, 1.498) | 0.157 |
| AIP (continuous) | - | 1.273 | (0.927, 1.747) | 0.135 |
| AIP |  |  |  |  |
| T1 | 199 / 7202 | 1 [Reference] | 1 [Reference] |  |
| T2 | 296 / 7203 | 1.026 | (0.831, 1.266) | 0.813 |
| T3 | 359 / 7199 | 1.134 | (0.899, 1.430) | 0.289 |

Note: Adjusted for gender, age, ethnic, economic level, BMI, hypertension, smoking status, energy intake and exercise status. Due to multiple comparisons being performed, both the P-values and the FDR-adjusted *P*-values were provided. Abbreviations: DII, Dietary Inflammatory Index; SIRI, System Inflammation Response Index; TyG, Triglyceride Glucose Index; AIP, Atherogenic index of plasma; BMI, body mass index; OR, Odds ratio; HR, Hazard ratio; CI, confidence interval; T, Tertile. ORs/HRs with statistical significance (P < 0.05) are presented in bold.

**Table S10 Inflammation-related indices mediate the association of metabolic indicators with CVD and mortality** **after multiple imputation**

| M/X | Outcome | TyG | AIP |
| --- | --- | --- | --- |
| **CVD prevalence** | | | |
| DII | ACME | 0.000086 (0.000085, 0.000088) *** | 0.00104 (0.00103, 0.00106) *** |
|  | ADE | 0.00351(0.00349, 0.00352) *** | 0.0412(0.0409, 0.0414) *** |
|  | Prop. Mediated(%) | 2.41% (2.36%, 2.45%)*** | 2.46% (2.43%, 2.51%) *** |
| SIRI | ACME | -0.000062 (-0.000063,-0.000061) *** | -0.00060(-0.00061,-0.00059) *** |
|  | ADE | 0.00361(0.00359,0.00363)*** | 0.0434(0.0431,0.0436)*** |
|  | Prop. Mediated(%) | -1.76%(-1.78%, -1.73%) *** | -1.40%(-1.42%, -1.37%)*** |
| **All-cause morality** | | | |
| DII | ACME | 1.013 (1.008, 1.020)*** | 1.0307 (1.0180, 1.0443)*** |
|  | ADE | 1.150 (1.071, 1.249)*** | 1.1714 (1.0146, 1.3606)* |
|  | Prop. Mediated(%) | 8.97% (4.59%, 17.95%)*** | 17.34% (8.14%, 63.32%)* |
| SIRI | ACME | 0.995 (0.990, 0.999)** | 0.991 (0.981, 0.999) * |
|  | ADE | 1.183 (1.101, 1.284)*** | 1.251 (1.077, 1.440) ** |
|  | Prop. Mediated(%) | -3.36 (-8.42%, -0.59%)** | -4.62% (-17.99%, -0.31%) * |
| **CVD morality** | | | |
| DII | ACME | 1.014 (1.006, 1.025)*** | 1.034 (1.016, 1.057)*** |
|  | ADE | 1.243 (1.067, 1.448)** | 1.225 (0.902, 1.628) |
|  | Prop. Mediated(%) | 6.80% (2.54%, 20.74%)** | 15.61% (-86.82%, 128.83%) |
| SIRI | ACME | 0.995 (0.988, 0.999)** | 0.991 (0.978, 0.999)* |
|  | ADE | 1.283 (1.101, 1.498)*** | 1.328 (0.987, 1.757) |
|  | Prop. Mediated(%) | -2.49% (-8.55%, -0.52%)** | -3.97% (-31.30%, 15.47%) |

Note: Adjusted for gender, age, ethnic, economic level, BMI, hypertension, smoking status, energy intake and exercise status. Abbreviations: CVD, Cardiovascular Disease; SIRI, System Inflammation Response Index; TyG, Triglyceride Glucose Index; AIP, Atherogenic index of plasma; BMI, body mass index; CI, confidence interval; ACME, average causal mediation effects (indirect effect); ADE, average direct effects. * P < 0.05, ** P < 0.01, and *** P < 0.001.

**Table S11 Joint associations of metabolic indicators and inﬂammation-related indices with CVD prevalence** **after multiple imputation**

| Index | Outcome | TyG | AIP |
| --- | --- | --- | --- |
| **CVD prevalence** | | | |
| DII | OR (95%CI), FDR-*P* | 1.62 (1.18, 2.23), 0.0033 | 2.12 (1.57, 2.86), <0.001 |
| SIRI | OR (95%CI) , FDR-*P* | 1.76 (1.35, 2.29), <0.001 | 2.25 (1.73, 2.92), <0.001 |
| **All-cause morality** | | | |
| DII | HR (95%CI) , FDR-*P* | 1.69 (1.40, 2.04), <0.001 | 1.63 (1.37, 1.94), <0.001 |
| SIRI | HR (95%CI) , FDR-*P* | 1.69 (1.34, 2.13) , <0.001 | 1.67 (1.37, 2.02), <0.001 |
| **CVD morality** | | | |
| DII | HR (95%CI) , FDR-*P* | 1.89 (1.29, 2.76), 0.0021 | 1.83 (1.28, 2.64), 0.0021 |
| SIRI | HR (95%CI) , FDR-*P* | 2.24 (1.30, 3.86), 0.0037 | 1.94 (1.25, 3.00), 0.0037 |

Note: ORs/HRs were adjusted for gender, age, ethnic, economic level, BMI, hypertension, smoking status, energy intake and exercise status. Due to multiple comparisons being performed, both the P-values and the FDR-adjusted *P*-values were provided. Abbreviations: CVD, Cardiovascular Disease; SIRI, System Inflammation Response Index; DII, Dietary Inflammatory Index; TyG, Triglyceride Glucose Index; AIP, Atherogenic index of plasma; BMI, body mass index; OR, Odds ratio; HR, Hazard ratio; CI, confidence interval; T, Tertile.
